# Twinkling Peptide Nanoemulsions Enable Precision Ultrasound Detection of Atherosclerotic Plaques

**DOI:** 10.1002/adfm.202415609

**Published:** 2024-12-13

**Authors:** Inhye Kim, Jacob C. Elliott, Atip Lawanprasert, Anthony M. Koehle, Grace M. Wood, Rita Castro, Julianna C. Simon, Scott H. Medina

**Affiliations:** Department of Biomedical Engineering, Pennsylvania State University, University Park, PA 16802-4400, USA; Graduate Program in Acoustics, Pennsylvania State University, University Park, PA 16802-4400, USA; Department of Biomedical Engineering, Pennsylvania State University, University Park, PA 16802-4400, USA; Department of Nutritional Sciences, Pennsylvania State University, University Park, PA 16802-4400, USA; Graduate Program in Acoustics, Pennsylvania State University, University Park, PA 16802-4400, USA; Department of Nutritional Sciences, Pennsylvania State University, University Park, PA 16802-4400, USA, Department of Pharmaceutical Sciences & Medicines, Faculty of Pharmacy, Universidade de Lisboa, Lisbon 1649-003, Portugal; Department of Biomedical Engineering, Pennsylvania State University, University Park, PA 16802-4400, USA, Graduate Program in Acoustics, Pennsylvania State University, University Park, PA 16802-4400, USA; Department of Biomedical Engineering, Pennsylvania State University, University Park, PA 16802-4400, USA, Huck Institutes of the Life Sciences, Pennsylvania State University, University Park, PA 16802-4400, USA

**Keywords:** atherosclerosis, doppler ultrasound, fluorine, nanoemulsion, peptide self-assembly

## Abstract

Non-invasive imaging modalities that identify rupture-prone atherosclerotic plaques hold promise to improve patient risk stratification and advance early intervention strategies. Here, phase-changing peptide nanoemulsions are developed as theranostic contrast agents for synchronous ultrasound detection and therapy of at-risk atherosclerotic lesions. By targeting lipids within atherogenic foam cells, and exploiting characteristic features of vulnerable plaques, these nanoemulsions preferentially accumulate within lesions and are retained by intraplaque macrophages. It is demonstrated that acoustic vaporization of intracellular nanoemulsions promotes lipid efflux from foam cells and generates echogenic microbubbles that provide contrast-enhanced ultrasound identification of lipid-rich anatomical sites. In Doppler mode, stably oscillating peptide nanoemulsions induce random amplitude and phase changes of the echo wave to generate transient color imaging features, referred to as ‘twinkling’. Importantly, acoustic twinkling is unique to these peptide emulsions, and not observed from endogenous tissue bubble nuclei, generating diagnostic features that offer unprecedented spatial precision of lesion identification in 3D.

## 1. Introduction

Atherosclerosis is a progressive inflammatory disease characterized by the accumulation of lipid- and immune cell-rich plaques in the arterial walls.^[1,2]^ Macrophages, antigen-presenting cells of the innate immune system, play a key role in all stages of atherosclerotic progression.^[3,4]^ For example, oxidized low-density lipoprotein (oxLDL) within nascent lesions creates pro-inflammatory complexes that promote the recruitment of monocyte-derived cells into the subendothelial space.^[5–7]^ These cells differentiate into macrophages that actively ingest oxLDL, generating lipid-rich foam cells with a limited capacity for migration.^[8]^ As a result, foamy macrophages persist within plaques and promote lipid storage, plaque growth, and progression of the lesion to a complex, pro-inflammatory phenotype. In this advanced stage, continued secretion of pro-inflammatory factors by foam cells, recruitment of other immune subsets and vascular cells, and eventual death of the cellular infiltrates, creates an iterative cycle that ultimately generates a pro-thrombotic necrotic core. These unstable plaques are then prone to rupture, creating high-risk pathological lesions that can potentiate lethal myocardial infarctions and ischemic stroke.^[9,10]^

Conventional diagnostic methods for atherosclerosis utilize intravascular ultrasound, X-ray angiography, magnetic resonance imaging, or optical coherence tomography to identify plaques based on morphologic lesion features and vessel stenosis.^[11–13]^ Pairing these tools to yield multi-modal modalities can enhance detection accuracy and enable physicochemical properties of the lesion to be predicted.^[14–16]^ Among these strategies, photoacoustic imaging, which pairs optical contrast and acoustic detection, has attracted significant attention due to its potential for label-free characterization of plaque burden. While these approaches provide a general anatomical assessment of lesion position, they lack the ability to distinguish stable from unstable plaques, and thus have limited resolution on disease pathology. Further, deep tissue imaging is often difficult for non-invasive modalities, and addressing this requires invasive intravascular procedures. Therefore, novel non-invasive methods that accurately identify high-risk lesions hold promise to improve patient risk stratification, reduce imaging-associated complications, and enable targeted therapeutic intervention before lethal plaque rupture.

**Figure 1.**
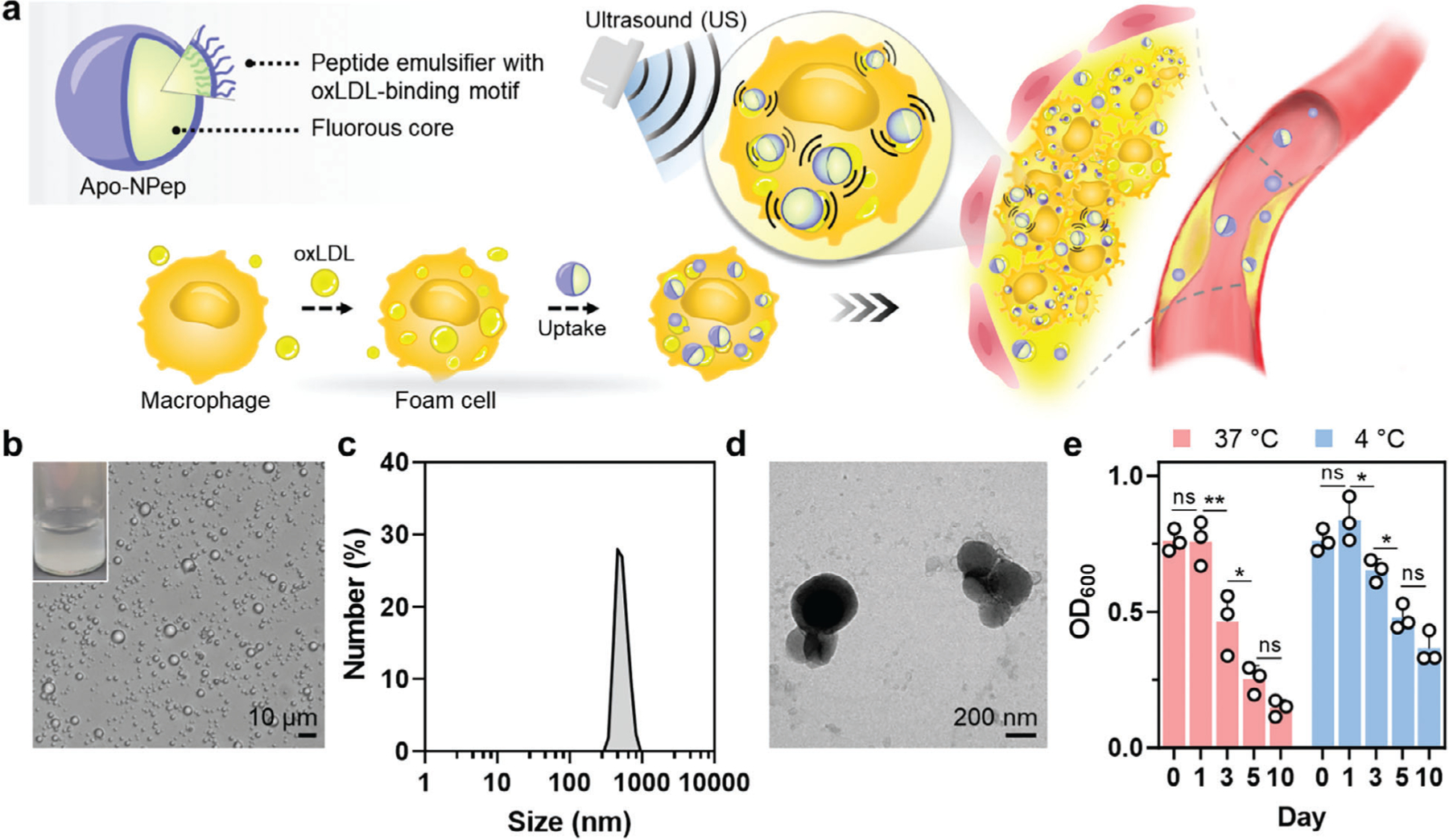
Design and biophysical analysis of oxLDL-targeting peptide nanoemulsions. a) Conceptual schematic of Apo-NPep architecture and mechanism of acoustic activation within intraplaque foam cells. b) Optical micrograph of Apo-NPep emulsions. Inset shows bulk dispersion. c) Size distribution and d) transmission electron micrograph of Apo-NPep emulsions. e) Time-dependent changes in optical density (OD_600_) of the emulsion solution at 37 °C (red) or 4 °C (blue). Initial concentration of emulsions at Day 0: ≈6 x 10^4^ emulsions mL^−1^. One-way ANOVA was used to determine statistical significance between indicated data sets; *n* = 3, ns: not significant, *p < 0.05, **p < 0.01.

Here, we report acoustically activated, phase-changing peptide nanoemulsions designed to selectively bind to oxLDL-rich foamy macrophages to provide contrast-enhanced ultrasound (US) diagnosis of vulnerable atherosclerotic plaques (Figure 1a). Preferential labeling is accomplished by exploiting the thin fibrous cap, characteristic of rupture-prone lesions, to enable intraplaque diffusion of the particles into at-risk anatomical sites. Rapid engulfment by resident foam cells, and selective binding to intracellular oxLDL, leads to retention of the emulsions within the pro-atherogenic cellular components. When stimulated by US, peptide nanodroplets undergo a liquid-to-gas phase transition that produces echogenic microbubbles in situ to serve as imaging nuclei. We show that, while this behavior provides contrast-enhanced B-mode imaging of lesions, under color Doppler US the peptide nanoemulsions exhibit a unique twinkling artifact that enhances the diagnostic precision of intraplaque foamy macrophage location in 3D tissues. Simultaneously, we find that US vaporization of peptide nanoemulsions stimulates efflux of oxLDL from foam cells, likely due to cavitation-induced mechanical fractionation of lipid droplets that facilitates transport of oxLDL to the cellular membrane. This suggests that, in addition to transforming the diagnosis of atherosclerotic lesions, this theranostic platform may simultaneously reduce lipid burden and shrink plaques. By leveraging the low-cost, portable, and non-ionizing nature of diagnostic US, this non-invasive modality provides real-time, continuous, and radiation-free theranosis of atherosclerotic arterial burden.

## 2. Results

### 2.1. Development and Characterization of Apo-NPep Emulsions

Atherosclerotic plaque-targeting peptide emulsions were prepared using the *de novo* designed peptide emulsifier: F_F_F_F_F_F_F_F_F_F_GDWFKAFYDKVAEKFKEAF (F_F_ = pentafluoro phenylalanine; see structure in Figure S1, Supporting Information). The C-terminal 18-mer (underlined in sequence) represents a reported peptide analogue of the Apolipoprotein A1 (Apo-A1), a primary component of high density lipoprotein, that specifically binds to oxidized lipids.^[17–19]^ This is conjugated to a fluorine-rich C-terminal tail that promotes interfacial assembly of the peptide emulsifier at the surface of a perfluorocarbon (PFC) nanodroplet to generate colloidally stable emulsions.^[20]^ A glycine spacer separates these two functional sequences to preserve the *α*-helical structure of the oxLDL-targeting motif, which is essential for its lipid-binding specificity. Mixing the peptide emulsifier with the PFC solvent perfluoropentane (PFP), and then sonicating the mixture with distilled water (see methods in Supporting Information), generated a dispersion of oxLDL-targeting peptide nanoemulsions (Figure 1b), hereafter referred to as Apo-NPeps, with an average diameter of ≈500 nm (Figure 1c,d). This size is ideal for cardiovascular labeling as it is sufficient to promote contact of Apo-NPep emulsions with the arterial wall, which sediment in hemodynamic flow due to the density of the PFP particle core (*ρ*_PFP_ = 1.6 g mL^−1^, *ρ*_blood_ = 0.9 g mL^−1^), while avoiding rapid renal clearance.^[14,21]^ Optical density measurements at 4 and 37 °C demonstrated that Apo-NPeps remain colloidally stable over the first day of storage (Figure 1e), and persisted in sufficient yield for imaging studies up to 10 days (47% and 26% remaining at 4 and 37 °C, respectively). Parallel particle characterization assays confirmed that Apo-NPeps retain their size over this storage period (Figure S2, Supporting Information).

### 2.2. Apo-NPep Targeting of Intraplaque oxLDL

**Figure 2.**
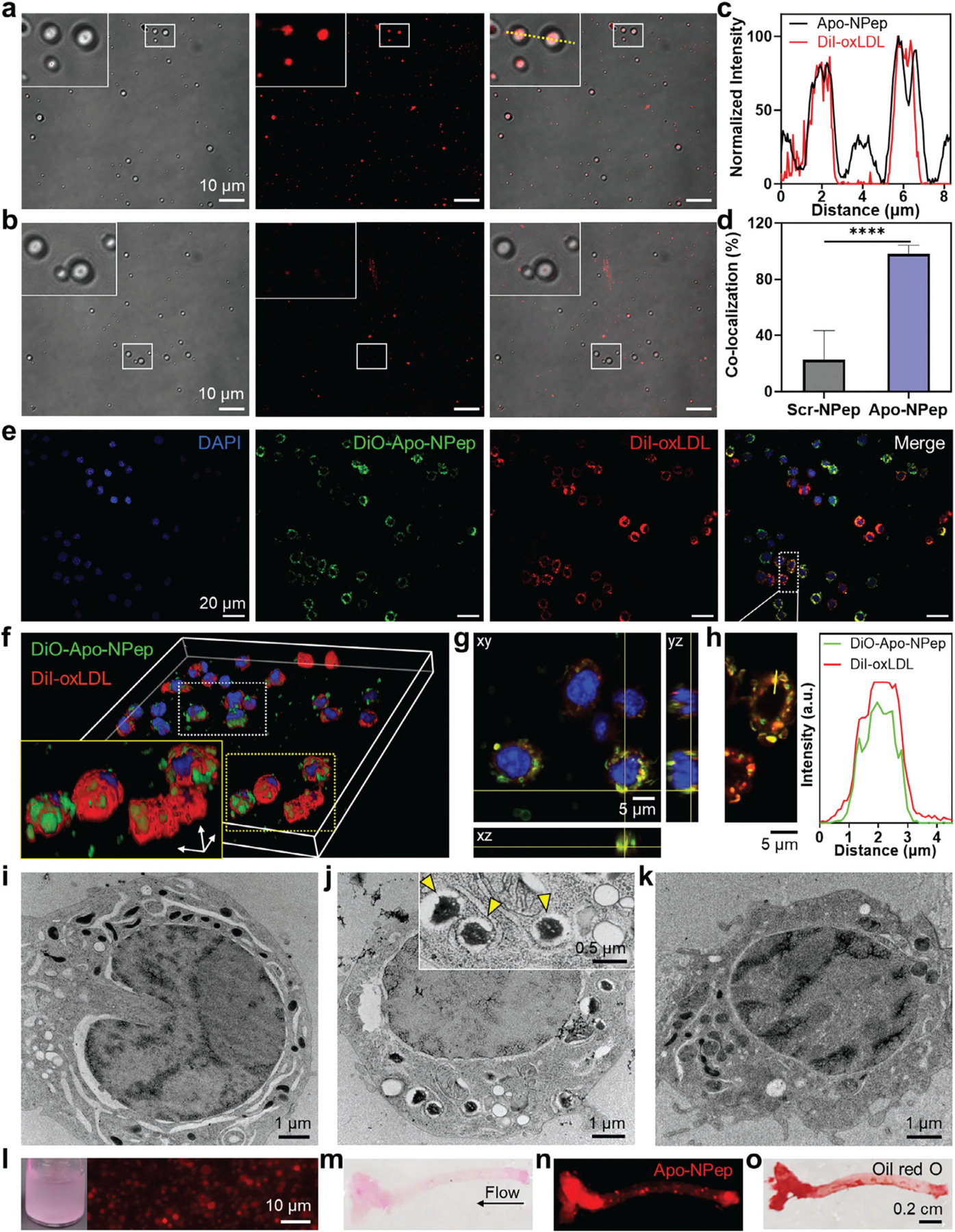
In vitro and ex vivo analysis of intraplaque Apo-NPep accumulation. a,b) Confocal laser scanning micrographs of (a) Apo-NPep and (b) Scr-NPep binding to DiI-labeled oxLDL; *left*: brightfield; *middle*: DiI-oxLDL; *right*: merged image. Insets show magnified region indicated by white boxes. c) Normalized intensity profile of Apo-NPep (black) and DiI-oxLDL (red) signals captured from the region in panel (a) indicated by the yellow dotted line. d) Percent co-localization of DiI-oxLDL and emulsion signals for Scr-NPep and Apo-NPep formulations; *n* = 276 particles analyzed from three experimental replicates. Unpaired *t*-test was used to determine statistical significance between indicated data sets; ^****^p < 0.0001. e) Confocal laser scanning micrographs of DiI-oxLDL loaded RAW 264.7 macrophages (red) treated with DiO-loaded Apo-NPep emulsions (green) for 6 h. DAPI (blue) indicates cell nuclei. f) 3D fluorescent projection of Apo-NPep phagocytosis into foamy macrophages. Inset shows magnified region identified by yellow dashed box. g) Magnified orthogonal projections identified by white dashed box in panel (f). h) Fluorescent intensity profile of Apo-NPep emulsions (green) and oxLDL (red) along yellow line in the selected confocal region shown to the left. i–k) Transmission electron micrographs of (i) untreated RAW 264.7 foamy macrophages, or cells treated with j) Apo-NPep or (k) Scr-NPep emulsions for 15 h. Electron dense oxLDL-droplets appear as dark circular/ellipsoidal structures. Yellow triangles in inset of panel (j) demonstrates oxLDL droplets enwrapped with Apo-NPep emulsions. l) Epifluorescent micrograph of DiI-loaded Apo-NPep emulsions used for ex vivo plaque accumulation assays. Inset shows dispersion of fluorescently labeled particles. m) Representative photograph of an aortic arch isolated from mice fed a high-fat diet and perfused with DiI-loaded Apo-NPep emulsions (pink color). n) Fluorescent whole tissue image of DiI-loaded Apo-NPep accumulation at atheroma sites. o) Photograph of atherosclerotic plaque staining in aorta tissue by Oil red O. Additional images of replicate ex vivo experiments can be found in Figure S11 (Supporting Information).

Next, we assessed the oxLDL-binding specificity of the emulsions using confocal laser scanning microscopy (Figure 2a–d). These studies include emulsions prepared from a scrambled version of the Apo-targeting peptide (Scr-NPep, see sequence in Figure S3, Supporting Information) to evaluate the specificity of Apo-NPep binding to oxLDL.^[22]^ Micrographs in Figure 2a,b demonstrate a clear co-localization of Apo-NPep with DiI-labeled oxLDL, with no such specificity observed for Scr-NPep controls. This suggests Apo-NPeps bind to oxLDL through specific ligand-target interactions, as opposed to non-specific adsorption to the surface of the lipid droplets. Spatially collated mapping of the Apo-NPep brightfield and DiI-oxLDL signals support this assertion (Figure 2c), with quantification of results demonstrating a 4.3-fold higher percentage of particle-oxLDL co-localization for Apo-NPep (98.0 ± 6.1%) relative to Scr-NPep (22.6 ± 20.8%) (Figure 2d).

We next compared the uptake of Apo- and Scr-NPeps into oxLDL-laden foamy macrophages and their ability to bind to intracellular lipid droplets. To rule out artifacts caused by cell toxicity, the biocompatibility of the emulsions were first assessed in RAW 264.7 macrophages after a 24 h incubation with Apo-NPep and Scr-NPep formulations (Figures S4–S6, Supporting Information). No statistically significant loss of RAW 264.7 viability was observed at the imaging compatible concentration of both particles. In parallel assays, we confirmed that treating RAW 264.7 macrophages with DiI-oxLDL (10 μg mL^−1^) for 4 h was sufficient to generate lipid-rich foam cells (Figure S7, Supporting Information). With these in vitro conditions optimized, we prepared fluorescently-labeled NPep emulsions and tracked their time-dependent internalization into foamy macrophages via flow cytometry (Figure S8, Supporting Information). Results demonstrated that maximum intracellular uptake of Apo-NPep occurred between 6 – 18 h of incubation and persisted within the macrophages for >24 h. Follow-up laser scanning confocal microscopy of oxLDL-laden foam cells demonstrated significant co-localization of Apo-NPeps with intracellular lipid droplets at both 6 h (Figure 2e) and 24 h (Figure S9, Supporting Information) of incubation. Serial confocal Z-stack images (Figure 2f,g) and fluorescence surface overlays (Figure 2h) further confirm the superposition of Apo-NPeps and oxLDL signals within foamy macrophages. Interestingly, TEM micrographs, shown in Figure 2i–k, demonstrate that Apo-NPep emulsions completely envelope oxLDL droplets within the macrophage lysosome, creating a coacervate-like internal structure (Figure 2j; additional images in Figure S10, Supporting Information). However, phagocytosed Scr-NPep controls do not show this same behavior (Figure 2k) and are not observed localized to intracellular oxLDL droplets. Intriguingly, Apo-NPeps appeared to shrink the size of internal oxLDL droplets and induce a sponge-like morphology (Figure S10, Supporting Information). This suggests Apo-NPeps may solubilize oxLDL to promote its removal from foamy macrophages,^[17]^ an assertion we confirm in later efflux studies. Conversely, intracellular oxLDL droplets in macrophages treated with Scr-NPeps appear morphologically similar to untreated controls, which corroborates later findings on the inability of Scr-NPeps to induce oxLDL efflux from foam cells. In sum, our results demonstrate the ability of Apo-NPeps to selectively target and bind oxLDL within atherogenic foamy macrophages and reveal unique nanoarchitectures formed by the emulsion-functionalized intracellular lipid droplets that hints at additional therapeutic functionality.

Encouraged by these findings, we investigated the ability of Apo-NPep emulsions to extravasate from the vasculature and accumulate within atherosclerotic lesions. We used the apolipoprotein E deficient (ApoE−/−) mouse, a well-characterized pre-clinical model that replicates all recognized stages of human atherosclerosis and accumulates aortic atheromas.^[23,24]^ Aortas from ApoE−/− mice fed an atherogenic high-fat diet^[25]^ were harvested and perfused with fluorescently labeled Apo-NPep formulations (Figure 2l). Whole tissue imaging confirmed the presence of Apo-NPeps in the aortic arch (Figure 2m,n), a region particularly prone to plaque accumulation due to low fluid shear stresses.^[26]^ This was then followed by perfusion of Oil red O (ORO) to stain the lipid content in atherosclerotic plaques.^[27]^ As expected, Apo-NPep signals were highly co-localized with ORO stained anatomical regions (Figure 2o), indicating selective accumulation of the emulsions within atherosclerotic lesions (see additional replicate images in Figure S11, Supporting Information). Control experiments confirmed Apo-NPep and ORO colocalization was not due to non-specific hydrophobic interactions between these components (Figure S12, Supporting Information), which could otherwise create artifactual staining. Taken together, our results demonstrate that Apo-NPeps translocate across the fibrotic vascular surface of atherosclerotic plaques,^[28]^ transport to the subendothelial space,^[14]^ and selectively accumulate within intraplaque foamy macrophages due to oxLDL binding.

### 2.3. Acoustic Activation and oxLDL Efflux Activity of NPep Emulsions

**Figure 3.**
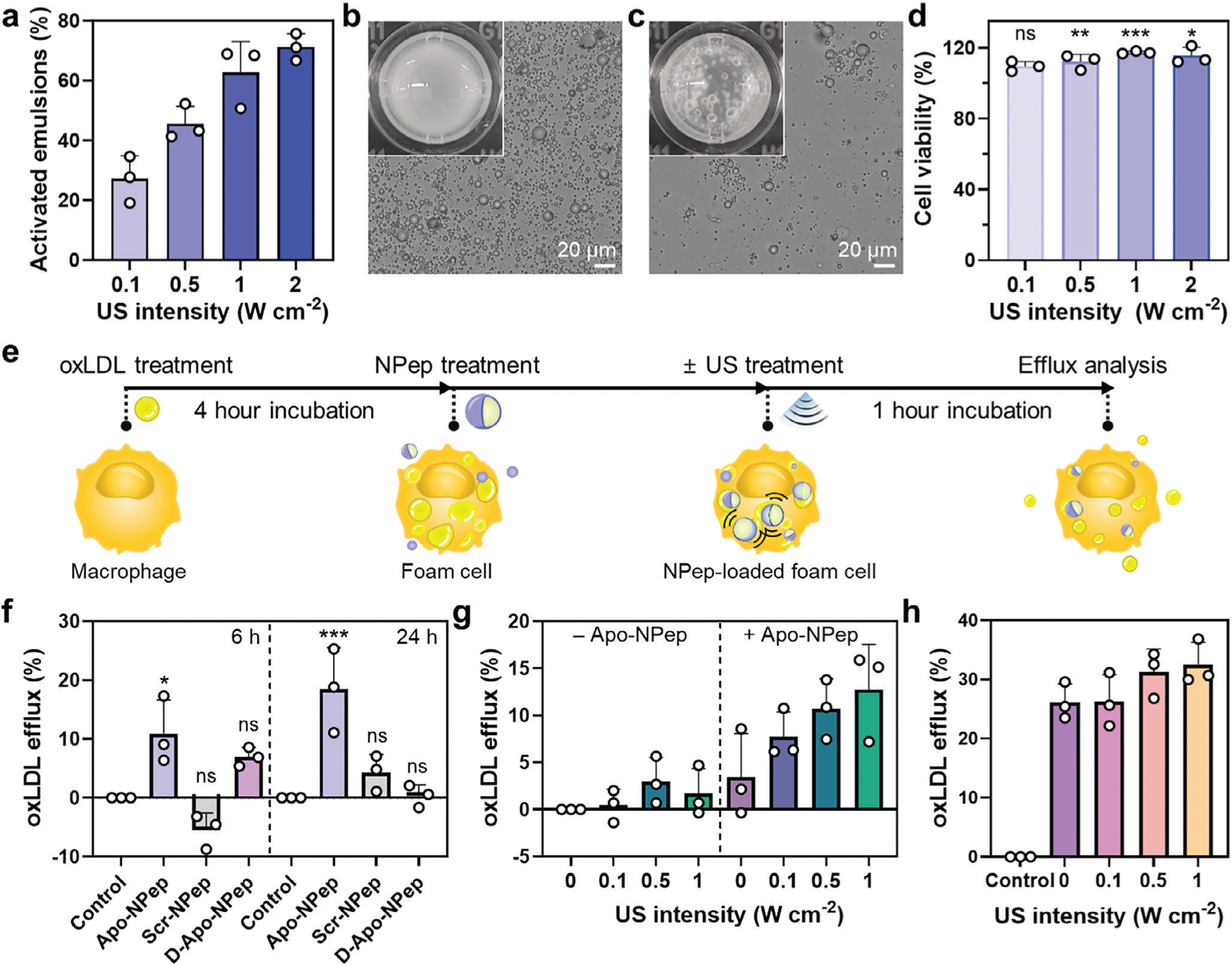
US activation and oxLDL efflux activity of Apo-NPep emulsions. a) Percent vaporized (activated) Apo-NPep emulsions at 37 °C as a function of US intensity (1 MHz, 50% duty cycle, 1 min. exposure, *n* = 3). US parameters employed are shown in Table S1 (Supporting Information). b,c) Representative optical images of Apo-NPeps (b) before and (c) after US activation (1 MHz, 2 W cm^−2^, 50% duty cycle, 1 min. exposure). Insets show Apo-NPep solution opacity (b) before and (c) after US treatment, demonstrating formation of coalescing bubbles that confirm Apo-NPep phase-change. d) Viability of Apo-NPep loaded RAW 264.7 cells following US exposure at the indicated intensity (1 MHz, 50% duty cycle, 1 min. exposure, *n* = 3). Unpaired *t*-tests determined statistical significance relative to controls exposed to corresponding acoustic intensity in the absence of emulsions; *p < 0.05, ^**^p < 0.01, ^***^p < 0.001. e) Schematic illustration of oxLDL efflux from foam cells following US activation of NPep emulsions. f) Percent oxLDL efflux from RAW 264.7 foam cells relative to control, following a 6 or 24 h treatment with the indicated NPep formulation. Untreated control efflux was set to 0% and used to normalize treatment results. One-way ANOVA was used to determine statistical significance relative to control; *n* = 3; ns: not significant, *p < 0.05, ***p < 0.001. g) Percent oxLDL efflux from foam cells in the absence (*left*) or presence (*right*) of internalized Apo-NPep emulsions activated at the indicated US intensity (*n* = 3). h) Percent oxLDL efflux from foam cells treated with Simvastatin-loaded Apo-NPep emulsions and activated at the indicated US intensity (*n* = 3) after 24-h incubation. Control is emulsion-untreated foam cells. US conditions used in panels (g,h) are 1 MHz, 50% duty cycle, 1 min. exposure at room tempearture.

Our previous findings show that Apo-NPep alters the morphology of oxLDL structures within foam cells and suggest that acoustic cavitation of the bound emulsions may mechanically fractionate the lipid droplets to promote their efflux. To test this assertion, we first investigated the acoustic sensitivity of the emulsions through optical density measurements (Figure 3a–c). Results indicate that the population of acoustically vaporized Apo-NPep particles increases monotonically with US intensity (Figure 3a), from 27% at 0.1 W cm^−2^ to >70% at 2 W cm^−2^. This acoustic activation of Apo-NPeps is due to a liquid-to-gas phase transition of the PFP core that occurs during the rarefaction (expansion) phase of the US waveform.^[29]^ Vaporization of the liquid nanodroplets to form microbubbles, a process referred to as cavitation,^[30]^ can be observed with the naked eye (see Figure 3b,c). Under these conditions, US activation of Apo-NPeps within treated RAW 264.7 cells did not compromise their viability (Figure 3d; Figure S13, Supporting Information).

Next, given the oxLDL destabilizing effects of the emulsions and the anti-atherogenic properties of the Apo-A1 mimetic peptide,^[18,19]^ we investigated the efflux of lipids from foamy macrophages treated with NPep emulsions. In addition to the therapeutic Apo-NPep formulation, we included scrambled emulsions (Scr-NPep) to compare their lipid efflux ability. Time-dependent oxLDL efflux from the foamy macrophages was evaluated via flow cytometry analysis of NPep-treated foam cells with and without US exposure (see experimental design in Figure 3e). Result in Figure 3f show that, in the absence of the US trigger, Apo-NPep emulsions stimulate greater oxLDL efflux from macrophages compared to Scr-NPep. Importantly, this efflux occurred without acoustic particle cavitation, indicating that binding of intact Apo-A1 mimetic emulsions to oxLDL is, alone, sufficient to reduce the lipid burden of foamy macrophages. This corroborates our prior TEM results showing shrinkage of lipid droplets within Apo-NPep treated macrophages before the application of US (Figure 2j; Figure S10, Supporting Information). This effect also increases with time, with oxLDL release from Apo-NPep treated cells increasing 1.7-fold between the 6- and 24-h incubation time points. As expected, Scr-NPep did not produce a statistically significant increase in lipid efflux relative to untreated controls. Additionally, emulsions prepared from the enantiomeric Apo-A1 peptide surfactant, referred to as D-Apo-NPep (Figure S14, Supporting Information), exhibited a reduced ability for lipid excretion compared to native Apo-NPep (Figure 3f). This was not expected given prior reports demonstrating that D-Apo-A1 peptides possess similar oxLDL efflux potency to their L-analogue.^[31,32]^ Our contrary findings are most likely due to the altered *α*-helicity of the oxLDL-binding motif in our D-Apo emulsifier relative to the native L-Apo sequence (Figure S15, Supporting Information), which is important given that secondary structure is reported to be a critical factor for the lipid binding and efflux activity of the peptide. Finally, US stimulation was found to further enhance oxLDL efflux from foam cells in an intensity-dependent manner, but only in the presence of Apo-NPep emulsions (Figure 3g), with no effect observed for Scr-NPep (Figure S16, Supporting Information). This is presumably due to mechanical fractionation of intracellular lipid droplets by the cavitating particles, which requires the emulsions to be closely bound to the oxLDL droplet surface. Notably, cavitation of Apo-NPep treated foam cells at the highest tested acoustic intensity (1.0 W cm^−2^) yielded a 3.7-fold increase in oxLDL efflux compared to non-insonated controls (0 W cm^−2^).

Encouraged by these results, we loaded Simvastatin, an FDA-approved anti-atherosclerotic drug, into Apo-NPep emulsions to test whether US-mediated delivery of the pharmacologic cargo would further promote lipid efflux (Figure 3h). Importantly, Simvastatin demonstrates pleiotropic effects in macrophages, including promoting the efflux of cholesterol stored within lysosomes.^[33]^ In the absence of the US trigger, Simvastatin-loaded Apo-NPep emulsions yielded a ≈8% improvement in oxLDL efflux from foamy macrophages after 24 h of treatment, relative to the non-drug loaded formulations (comparison of Figure 3h (+Simvastatin, 0 W cm^−2^) to Apo-NPep results in Figure 3f (-Simvastatin)). This suggests that Simvastatin may gradually diffuse out of the emulsions over 24 h to enhance efflux activity in Apo-NPep treated foam cells. US activation of Simvastatin-loaded Apo-NPeps yielded improved results, with ≈32% removal of total intracellular lipid burden from Apo-NPep treated foam cells activated at the highest tested US intensity (1 W cm^−2^, see Figure 3h). Collectively, our findings demonstrate that Apo-NPeps can execute synergistic biochemical (lipid-binding and solubilizing), mechanical (US cavitation) and pharmacologic (statin release) effects to stimulate rapid and efficient clearance of lipids from atherogenic foam cells.

### 2.4. US Imaging of Apo-NPep Treated Foamy Macrophages in Tissues

**Figure 4.**
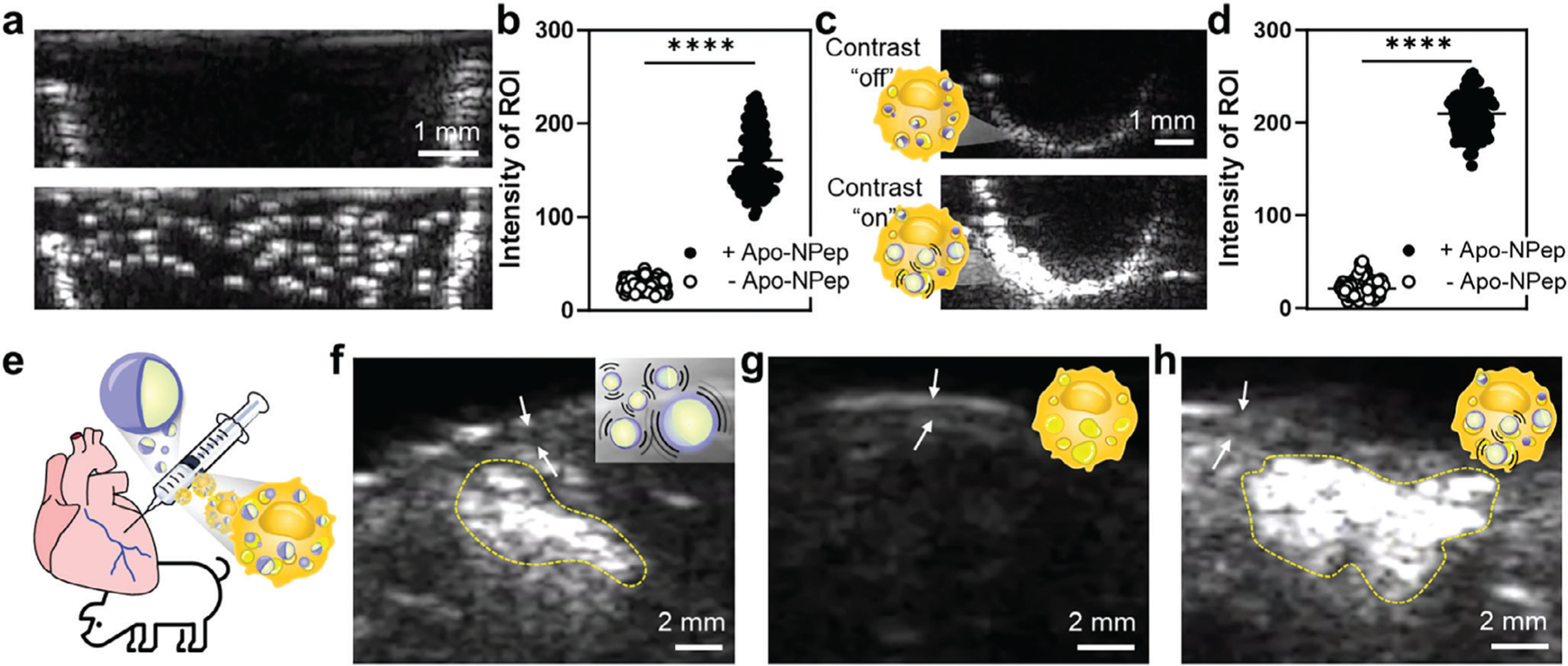
B-mode US imaging of Apo-NPep emulsions. a) Representative B-mode images of Apo-NPep emulsions in an agar phantom before (*top*) and after (*bottom*) US-mediated particle cavitation (vaporization occurs at 18.5 MHz, p+ = 1.0 MPa, p− = 0.6 MPa). b) Quantification of B-mode signals from the emulsions (+Apo-NPep) or background control (-Apo-NPep). Unpaired *t*-test determined statistical significance; *n* = 110, ^****^p < 0.0001. c) Representative B-mode images of Apo-NPep loaded RAW 264.7 foam cells in agar phantoms before (*top*, p+ = 0.1 MPa, p− = 0.1 MPa) and after (*bottom*, p+ = 1.0 MPa, p− = 0.6 MPa) emulsion vaporization using an 18.5 MHz US transducer. d) Quantification of B-mode signals from non-labeled foam cells (-Apo-NPep) and Apo-NPep labeled foam cells (+Apo-NPep). Unpaired *t*-test determined statistical significance; *n* = 100, ****p < 0.0001. e) Schematic illustration of ex vivo injection of Apo-NPep emulsions or Apo-NPep-foam cells to create model lesions adjacent to the porcine coronary artery. f–h) Representative B-mode images of injected depots containing (f) free Apo-NPep emulsions, (g) non-labeled RAW 264.7 foam cells, and (h) Apo-NPep labeled foam cells. B-mode US conditions for panels (f–h): 5.2 MHz, p+ = 1.9 MPa, p− = 0.7 MPa. White arrows identify the lumen of the adjacent coronary artery.

The imaging potential of the emulsions was first evaluated by loading Apo-NPeps into tissue-mimetic agar phantoms and collecting B-mode US signals (Figure 4a). Once exposed to US (18.5 MHz; p+ = 1.0 MPa, p− = 0.6 MPa), Apo-NPeps generated echogenic microbubbles that could be clearly resolved from the background, producing a 5.8 signal-to-noise ratio (Figure 4b). This result confirms the rapid phase-shift of emulsions into bubbles within the acoustic field, further supported by video recordings showing the buoyancy of the cavitating contrast agents (Movie S1, Supporting Information). Additional studies confirmed that B-mode signal intensity is proportional to emulsion concentration (Figure S17, Supporting Information), and that the particles can be stored for up to 5 days at 4 °C without a significant loss of imaging performance (Figure S18, Supporting Information). Next, we investigated the persistence of Apo-NPep signals following uptake of the emulsion into foam cells. Remarkably, B-mode imaging of Apo-NPep-loaded foamy macrophages, hereafter referred to as Apo-NPep-foam cells, showed a further enhancement in the contrast of the emulsion imaging nuclei, producing a 9.2-fold enhancement in signal-to-noise (Figure 4c,d; additional replicate images in Figure S19a,b, Supporting Information). Control foam cells, void of intracellular Apo-NPep contrast agents, were acoustically invisible (Figure S19c,d, Supporting Information). The increase in B-mode contrast of Apo-NPep-foam cells relative to the free Apo-NPep particles is likely due to accumulation of multiple emulsions within each cell. In fact, some cells had a sufficient intracellular load of the Apo-NPep emulsions that, following US conversion of the particles into microbubbles, the foamy macrophages became buoyant (Movie S2, Supporting Information). This result hints at the potential for these emulsions to promote migration of foam cells out of atherosclerotic plaques, where US vaporization of phagocytosed Apo-NPep may lead to cellular buoyancy that encourages their localization to the plaque-endothelial interface.

Encouraged by the promising acoustic imaging capabilities of our platform, we next validated the performance of free Apo-NPep emulsions and Apo-NPep-foam cells in ex vivo tissues, using a porcine heart as an exemplary model (Figure 4e). To generate a facsimile of pathologic lesions, depots of free emulsions, or foamy macrophages loaded without/with Apo-NPep emulsions, were injected adjacent to a coronary vessel. B-mode imaging was then performed using a 5.2 MHz transducer, a frequency selected to ensure moderate tissue penetration of the acoustic signals and to match traditional frequencies of clinical cardiac ultrasonography (4 – 7 MHz).^[34]^ When free Apo-NPep emulsions were injected, a significant enhancement in B-mode grayscale contrast was observed (see yellow dashed region in Figure 4f), confirming the contrast agents were active in physiologic tissues. Similar to the results from our agar phantom experiments, Apo-NPep-foam cells produced the most intense imaging features, and therefore yielded the greatest contrast, while foamy macrophages without Apo-NPeps were acoustically silent (Figure 4g,h). Importantly, the improved contrast of Apo-NPep-foam cells allowed us to clearly resolve the boundaries of the model lesion and precisely identify its spatial localization relative to the arterial wall.

### 2.5. Doppler Twinkling of Apo-NPep Emulsions

**Figure 5.**
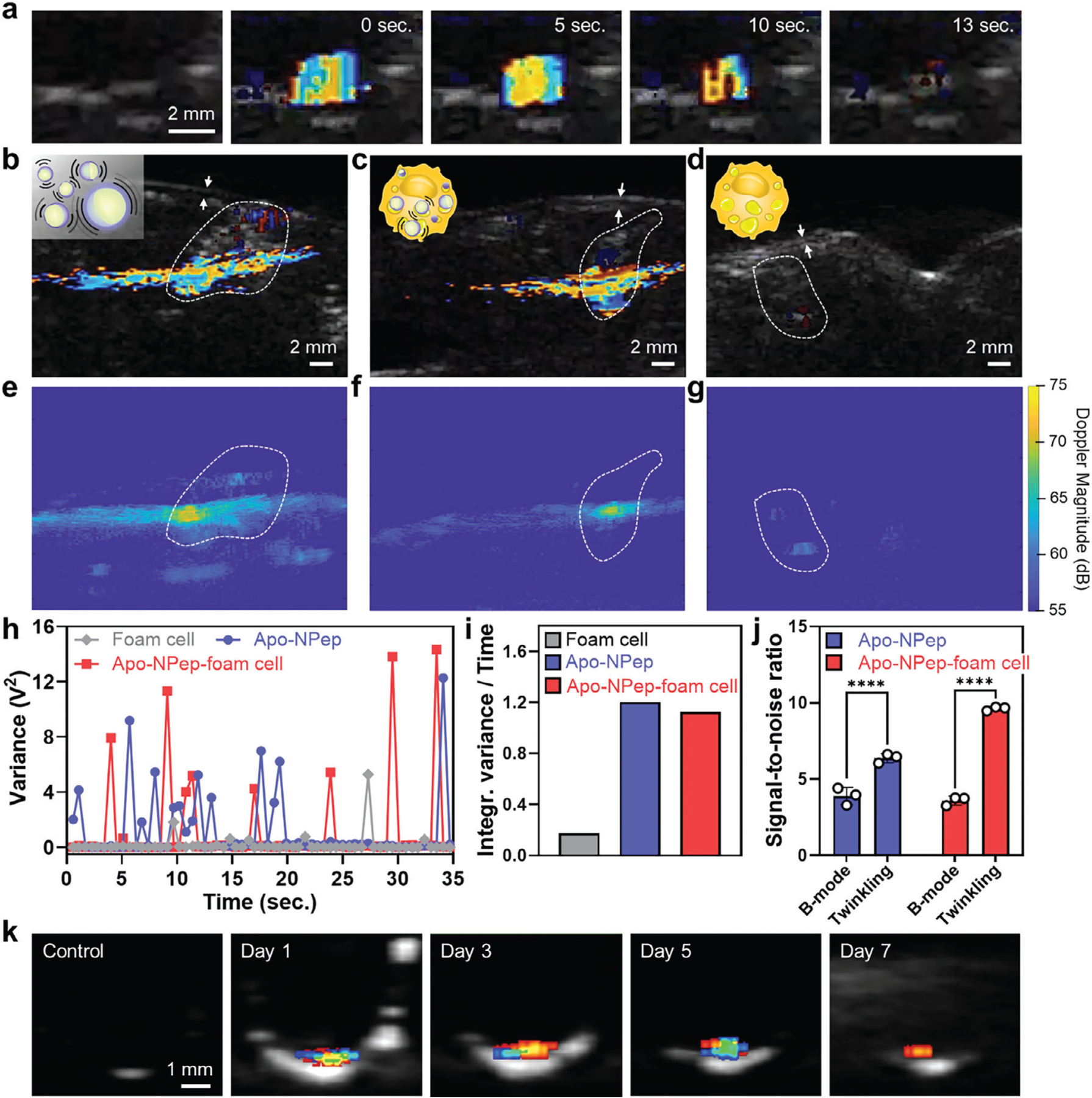
Apo-NPep Doppler twinkling. a) Color Doppler signals, superimposed on B-mode images, from Apo-NPep emulsions insonated in an agar phantom (Doppler conditions: 5.2 MHz, p+ = 3.8 MPa, p− = 1.6 MPa, color priority: 180, color persistence: 81, and Doppler power threshold: 0.26). Far-left panel displays the initial B-mode image without Doppler. Remaining panels show Doppler twinkling as a function of exposure time. b–d) Doppler twinkling in porcine heart tissue after delivery of (b) free Apo-NPep emulsions, (c) Apo-NPep labeled foam cells, and (d) non-labeled foam cells. Doppler imaging conditions: 5.2 MHz, p+ = 4.0 MPa, p− = 1.8 MPa, color priority: 255, color persistence: 0, and Doppler power threshold: 0.20. White dashed region of interest (ROI) indicates model anatomical lesion. White arrows identify coronary artery lumen. B-mode US imaging conditions used in panels (a–d): 5.2 MHz, p+ = 2.6 MPa, p− = 0.9 MPa. e–g) Spatial Doppler magnitude corresponding to panels (b–d), respectively. h) Time-dependent Doppler magnitude over a 35 s imaging window for non-labeled foam cells (gray), free Apo-NPep emulsions (blue), and Apo-NPep-foam cells (red). i) Integrated variance (area under the curve) of time-locked Doppler signals from panel (h). j) Calculated signal-to-noise ratio for Apo-NPep (blue) and Apo-NPep-foam cells (red) during B-mode and Doppler Twinkling imaging. Two-way ANOVA was used to determine statistical significance; *n* = 3, ****p < 0.0001. k) Persistance of twinkling features in Apo-NPep labeled foam cells over 7 days in culture (Doppler conditions: 6.5 MHz, p+ = 2.2 MPa, p− = 1.8 MPa, B-mode conditions: 7.5 MHz, mechanical index: 0.7).

While Apo-NPep-foam cell lesions can be identified from B-mode images, scattering of the acoustic signal in deep tissues may reduce feature resolution, increase the frequency of false positive signals, and collectively diminish the accuracy of our platform. This pitfall is shared by all current US contrast agents, and is caused by background B-mode speckle.^[35]^ However, during our US imaging experiments we discovered a unique imaging feature of Apo-NPeps that overcomes this limitation. When performing color Doppler US, we observed rapid color changes, referred to as acoustic ‘twinkling’, from the emulsions as they phase transitioned into echogenic microbubbles in agar phantoms (Figure 5a; additional replicate images in Figure S20, Movie S3, Supporting Information). Here, twinkling arises from random amplitude and phase changes of the US echo wave due to reflections caused by the impedance mismatch between the bubble and surroundings,^[36,37]^ generating transient Doppler signals. The wide spectral bandwidth of Apo-NPep twinkling is not shared by endogenous tissue and so these twinkling features are specific to our contrast agents. This phenomenon allowed us to resolve depots of Apo-NPep emulsions (Figure 5b; Movie S4, Supporting Information) and Apo-NPep-foam cells (Figure 5c; Movie S5, Supporting Information) more clearly in porcine tissue under Doppler imaging, relative to B-mode signals (Figure 4f–h), with precise spatial localization. Like B-mode imaging, foamy RAW 264.7 cells without Apo-NPep contrast agents are acoustically invisible in Doppler mode (Figure 5d; Movie S6, Supporting Information). Collating the Doppler magnitude within the imaging frame further resolved the focal point of the twinkling features and improved the spatial precision of lesion identification (Figure 5e–g).

To quantitively compare the occurrence of twinkling among samples, we plotted the time-dependent variance in Doppler signals from control foam cells, free emulsions (Apo-NPep), and emulsion-loaded foam cells (Apo-NPep-foam cells). Results in Figure 5h demonstrate more frequent Doppler twinkling events from the free emulsions compared to Apo-NPep-foam cells over the 35 s collection time. It is worth mentioning that the extended twinkling features from the Apo-NPep in tissues compared to its shorter duration (≈10 s) in the agar cavity (Figure 5a) may result from a high local concentration of particles within the tissues, which limits the displacement of emulsions in the acoustic field.^[38]^ Further, the reduced twinkling frequency of Apo-NPep-foam cells compared to the free emulsions may be due, in part, to viscoelastic dampening of cavitating microbubbles by the molecularly crowded intracellular environment.^[39,40]^ However, condensing the time-locked variance into a single integrated value demonstrates equivalent cumulative Doppler intensity from free emulsions and Apo-NPep-foam cells (Figure 5i). This suggests that the decreased frequency of emulsion twinkling events inside foamy macrophages is compensated by the higher loading of particles within each cell, leading to a higher per-event Doppler intensity for Apo-NPep-foam cells relative to emulsions alone. As a result, Doppler twinkling offers superior spatial contrast for lesion identification compared to B-mode imaging, with a 1.6- and 2.7-fold enhancement in the signal-to-noise ratio between the imaging modalities when using Apo-NPep and Apo-NPep-foam cells, respectively (Figure 5j). Moreover, Apo-NPeps were observed to persist in labeled macrophages and provide Doppler twinkling features for over 7 days (Figure 5k; Figure S21, Supporting Information). Finally, Apo-NPep emulsions stored intact for more than 2 months at 4 °C still produced twinkling features when imaged (Figure S22, Supporting Information), demonstrating an extraordinary stability relative to the minute-hour persistence of traditional microbubble contrast agents. Collectively, these findings demonstrate that the distinct Doppler twinkling behavior of Apo-NPeps can delineate atherosclerotic lesions within tissues with unprecedented spatial precision, and provide stable, multi-day imaging persistence utilizing standard clinical US hardware.

**Figure 6.**
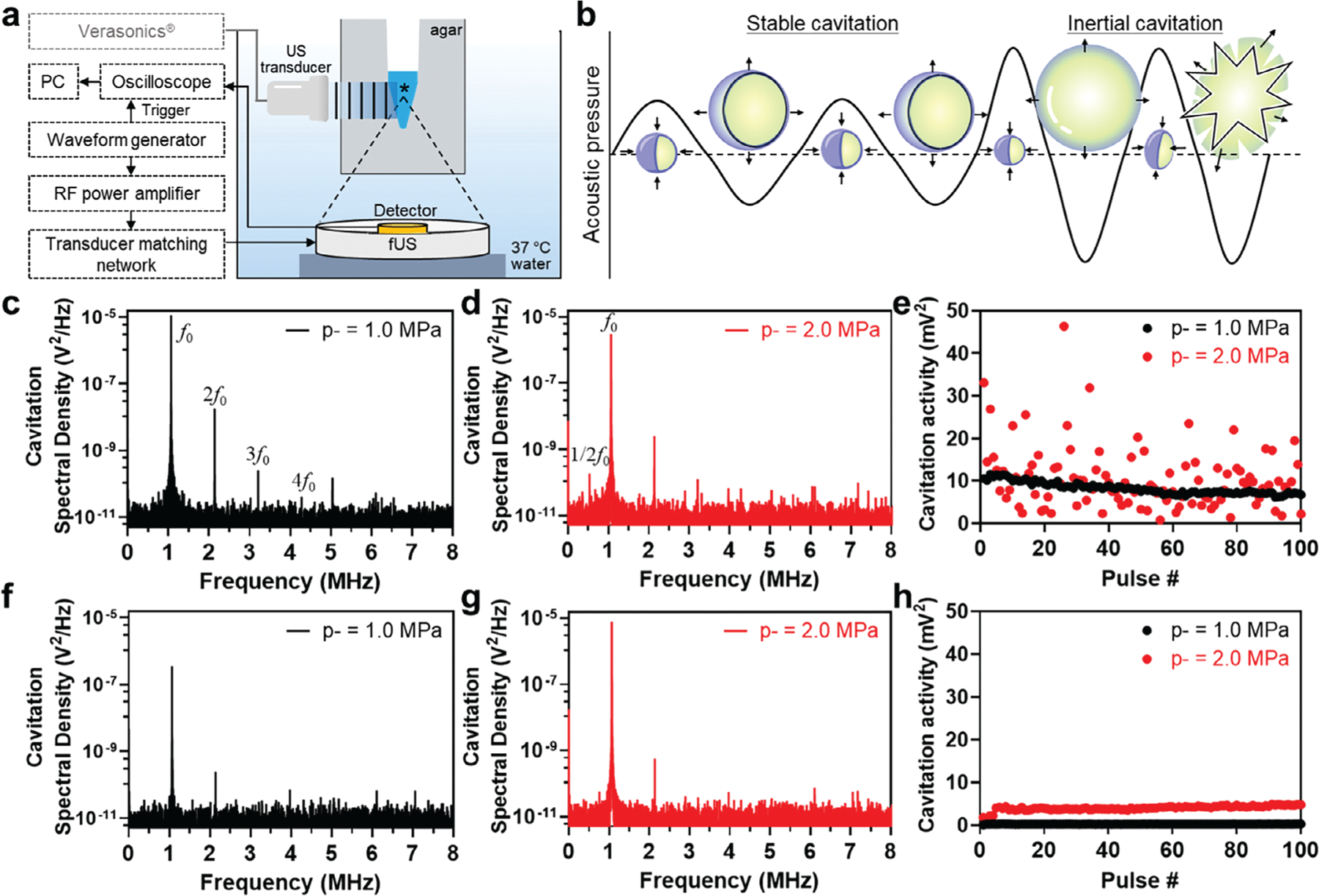
PCD analysis of Apo-NPep cavitation. a) A schematic of the PCD experimental setup using a 1.07 MHz fUS transducer. The asterisk (*) denotes the focal zone in the sample solution (blue). b) Conceptual diagram of stable and inertial cavitation of vaporized Apo-NPeps. c,d) Frequency spectra of oscillating Apo-NPep emulsions at c) intermediate (p+ = 1.3 MPa, p− = 1.0 MPa) and d) high (p+ = 2.5 MPa, p− = 2.0 MPa) acoustic pressures. e) Cavitation activity plots of Apo-NPep over 100 pulses at the indicated acoustic pressure. f–h) Frequency spectra and cavitation activity plots from degassed control solutions insonated under similar acoustic conditions.

Finally, to mechanistically probe the phase of cavitation responsible for Apo-NPep Doppler twinkling, we measured cavitation-induced acoustic signals from emulsions loaded into an agar phantom using a 1.07 MHz focused ultrasound (fUS) activating transducer and a focused passive cavitation detection (PCD) hydrophone (Figure 6a).^[41]^ By interpreting the harmonic periodicity and noise of the echo frequency spectra we can differentiate stable cavitation, where microbubbles undergo pulsatile volumetric changes at their equilibrium radius,^[35]^ from inertial cavitation, where bubbles oscillate out of equilibrium and collapse (Figure 6b).^[42]^ For this study, we monitored the cavitation activity of the emulsions at an intermediate (p+ = 1.3 MPa, p− = 1.0 MPa) and high (p+ = 2.5 MPa, p− = 2.0 MPa) acoustic pressure (Figure 6c–e). Degassed PBS was analyzed as a control (Figure 6f–h). Cavitation spectral density plots recorded at p− = 1.0 MPa showed harmonic echo frequencies from the activated emulsions at ≈2.1, 3.2, and 4.3 MHz (fundamental frequency *f*_0_ = 1.07 MHz) (Figure 6c). Increasing acoustic pressure to p− = 2.0 MPa yielded a subharmonic frequency at ≈0.5 Hz (1/2*f*_0_) (Figure 6d). The appearance of harmonic and subharmonic frequencies, as well as lack of broadband acoustic emission,^[43]^ suggests vaporized Apo-NPeps stably cavitate in the acoustic field.^[42,44]^ Interpreting the pulse-locked cavitation activity over 100 cycles demonstrates a stable cavitation signal at 11.5 mV^2^ for Apo-NPep particles activated at p− = 1.0 MPa (Figure 6e) which is >50 times higher than the control (≈0.2 mV^2^, Figure 6h). The subtle decline in cavitation activity for Apo-NPep particles over the 100 pulse window may be due to fluidic transport of the bubbles out of the focal region in the agar cavity, which was observed during B-mode imaging (Figure S23a,b, Movie S7, Supporting Information).^[41]^ Conversely, at p− = 2.0 MPa a large fluctuation in cavitation activity was observed (Figure 6e), which corresponds to a disappearance of the hyperechoic region in B-mode imaging captured during fUS-treatment (Figure S23c, Movie S8, Supporting Information). This is most likely due to the oscillating bubbles migrating away from the focal region in the agar cavity under increased acoustic pressures, rather than bubble collapse, as evidenced by the absence of broadband noise (Figure 6d). Taking our PCD results in conjunction with our Doppler imaging data, these findings suggest that twinkling features appear at intermediate acoustic amplitudes (p− = 1.6–1.8 MPa) and originate from stable cavitation of the Apo-NPep bubble.^[45]^ Importantly, this indicates that Apo-NPep bubble nuclei may be potentially recondensed back to an emulsion after US sonication to enable reuse of the particles over multiple imaging cycles,^[46]^ therefore significantly extending the persistence of the twinkling contrast agent in tissues.

## 3. Conclusion

A characteristic feature of advanced atherosclerosis is the substantive accumulation of lipid-engulfing macrophages in the arterial intima. Accordingly, contrast agents that can exploit this characteristic monocyte infiltration, in combination with the thin fibrous cap and persistent pro-inflammatory state of at-risk plaques, can improve lesion diagnosis and patient management. To address this need, we have developed nanoemulsion contrast agents that selectively target, and are internalized by, foam cells in atheromas to selectively label plaques for contrast-enhanced US diagnosis. It is worth noting that this strategy circumvents the limitations of conventional microbubble contrast agents, which are generally too large to efficiently translocate across the fibrous lesion surface. Additionally, the short half-lives of microbubbles in serum (≈2–20 min)^[47]^ are considered too short to provide the imaging time frame necessary for longitudinal monitoring of lesion morphology and burden. Conversely, we demonstrate that Apo-NPep Doppler twinkling can persist over multiple days to provide precise localization of atherogenic anatomical sites and enable long-term monitoring. Additional therapeutic functionality of the nanoemulsions to promote lipid efflux suggests they can stimulate plaque regression to revert at-risk lesions into stable phenotypes. Combining this theranostic modality with additional anatomic- and hemodynamic disease assessment may open opportunities for non-invasive investigation of plaque composition and lesion pathophysiology, together reducing patient morbidity and improving clinical outcomes.

## Supplementary Material

Supplementary Information

Movie S1

Movie S2

Movie S3

Movie S4

Movie S5

Movie S6

Movie S8

Movie S7

Supporting Information is available from the Wiley Online Library or from the author.

## Data Availability

The data that support the findings of this study are available from the corresponding author upon reasonable request.
