# Supplementary material for: Twinkling Peptide Nanoemulsions Enable Precision Ultrasound Detection of Atherosclerotic Plaques
Source: Adv Funct Mater. Author manuscript; Available in PMC 2025 Aug 28. (PMC12381953; doi:10.1002/adfm.202415609)
Supplement: Supplementary Information [file NIHMS2078927-supplement-Supplementary_Information.docx]

Supporting Information

**Twinkling Peptide Nanoemulsions Enable Precision Ultrasound Detection of Atherosclerotic Plaques**

*Inhye Kim, Jacob C. Elliott, Atip Lawanprasert, Anthony M. Koehle III, Grace M. Wood, Rita Castro, Julianna C. Simon, and Scott H. Medina**

**Content Page Number**

[**Materials and Experimental Methods** 3](#_Toc181397977)

[**Table S1.** US parameters employed 11](#_Toc181397978)

[**Figure S1.** Molecular structure of Apo-peptide emulsifier and its HPLC trace and mass spectrum 12](#_Toc181397980)

[**Figure S2.** Time-dependent change of Apo-NPep size over 10 days 13](#_Toc181397981)

[**Figure S3.** Molecular structure of Scr-peptide emulsifier and its HPLC trace and mass spectrum 14](#_Toc181397982)

[**Figure S4.** Viability of RAW 264.7 cells after 24 hours of incubation with emulsions 15](#_Toc181397983)

[**Figure S5.** Live/dead assay using RAW 264.7 cells treated with Apo-NPep 16](#_Toc181397984)

[**Figure S6.** Live/dead assay using RAW 264.7 cells treated with Scr-NPep 17](#_Toc181397985)

[**Figure S7.** Epifluorescence micrographs of RAW 264.7 cells incubated with DiI-oxLDL 18](#_Toc181397986)

[**Figure S8.** Time-dependent internalization of Apo-NPep emulsions into RAW 264.7 foam cells 19](#_Toc181397987)

[**Figure S9.** Confocal laser scanning micrographs of RAW 264.7 cells treated with DiI-oxLDL following incubation with DiO-loaded Apo-NPep emulsions 20](#_Toc181397988)

[**Figure S10.** TEM images of RAW 264.7 foam cells incubated with Apo-NPep emulsions 21](#_Toc181397989)

[**Figure S11.** *Ex vivo* analysis of intraplaque accumulation of Apo-NPep emulsions 22](#_Toc181397990)

[**Figure S12.** Microscopic analysis of interaction between Apo-NPep and ORO 23](#_Toc181397991)

[**Figure S13.** Optical images of Apo-NPep-treated RAW 264.7 cells following insonation 24](#_Toc181397992)

[**Figure S14.** Molecular structure of D-Apo peptide emulsifier and its HPLC trace and mass spectrum 25](#_Toc181397993)

[**Figure S15.** CD spectra of Apo- and D-Apo peptide emulsifiers in water 26](#_Toc181397994)

[**Figure S16.** oxLDL efflux from RAW 264.7 foam cells following US-activation of internalized Scr-NPep or Apo-NPep 27](#_Toc181397995)

[**Figure S17.** Increase in B-mode US signals of Apo-NPep as a function of emulsion concentration 28](#_Toc181397996)

[**Figure S18.** US imaging performance of Apo-NPep emulsions over 10 days 29](#_Toc181397997)

[**Figure S19.** B-mode images of RAW 264.7 foam cells unloaded or loaded with Apo-NPep 30](#_Toc181397998)

[**Figure S20.** Color Doppler US images of agar phantom loaded with Apo-NPep emulsions 31](#_Toc181397999)

[**Figure S21.** Optical micrographs of RAW 264.7 cells incubated with Apo-NPep for 1 – 7 days. 32](#_Toc181398000)

[**Figure S22.** Color Doppler US image of >2-month-old Apo-NPep emulsions 33](#_Toc181398001)

[**Figure S23.** B-mode images of agar phantom loaded with Apo-NPep captured during PCD study 34](#_Toc181398002)

[**References** 35](#_Toc181398003)

# **Materials and Experimental Methods**

**Materials:** Rink amide ProTide resin (LL) (100–200 mesh), oxyma pure, and all fluorenylmethoxycarbonyl (Fmoc) protected amino acids except Fmoc-pentafluoro-L-phenylalanine were purchased from CEM. Tris and ethylenediaminetetraacetic acid (EDTA) were obtained from VWR. Bovine serum albumin (BSA), sodium chloride (NaCl), and agar powder were obtained from Fisher Scientific. LC-MS grade water, acetonitrile, and formic acid (FA), dimethyl sulfoxide (DMSO) (bioreagent), dichloromethane (DCM), Slide-A-Lyzer dialysis cassettes (3.5kDa molecular weight cutoff, MWCO), diethyl ether, ProLong Diamond antifade mountant with 4’,6-diamidino-2-phenylindole (DAPI), sodium azide, low density lipoprotein (LDL) from human plasma, oxidized, 1,1’-dioctadecyl-3,3,3’,3’-tetramethylindocarbocyanine (DiI) conjugate (DiI-oxLDL), and oxidized LDL (oxLDL) were purchased from Thermo Fisher Scientific. In general, commercial oxLDL and DiI-oxLDL were used; however, as required, reagents were prepared according to literature protocols, with slight modifications.^[1]^ In brief, oxLDL was prepared by oxidizing commercial LDL using copper sulfate. LDL (2.5 mg mL^-1^) was first dialyzed (3.5 kDa MWCO) against phosphate buffered saline (PBS, 1x) for 2 hours to remove EDTA, and then incubated with Cu^2+^ (200 μM) at 37 °C overnight. Excess Cu^2+^ was subsequently removed by dialysis (3.5 kDa MWCO) against PBS for 2 hours. The oxLDL was spin-filtered (0.2 μm) prior to use. To prepare DiI-oxLDL, DiI (40 μg mL^-1^) in DMSO was mixed with oxLDL (0.39 mg mL^-1^) in PBS overnight at 37 °C in dark. Unbound dye was removed by ultrafiltration (Vivaspin^®^ 500, 10kDa MWCO) at 400 *g* for 6 hours at 4 °C, with periodic buffer replenishment (10 mM Tris, 150 mM NaCl, 0.3 mM EDTA, 2 mM sodium azide, pH 8.3). The conjugate was further dialyzed against the same buffer at 4 °C overnight. Before use, the final product was spin-filtered through a 0.2 μm membrane. *N*,*N*-Diisoproylcarbodiimide (DIC), trifluoroacetic acid (TFA), piperidine, Fmoc-pentafluoro-L-phenylalanine, hexafluoroisopropanol (HFIP), and triisopropylsilane (TIPS) were purchased from Chem-Impex. *N*,*N*-Dimethylformamide (DMF) and DMSO (spectrophotometric grade) were purchased from Alfa Aesar. Perfluorohexane (PFH) was purchased from Oakwood Chemicals. Perfluoropentane (PFP) was purchased from SynQuest Laboratories. PBS without calcium and magnesium, fetal bovine serum (FBS), 0.25% trypsin-EDTA (1x), and trypan blue solution (0.4%, w/v) in PBS were obtained from Corning. Thiazolyl blue tetrazolium bromide (MTT), propidium iodide (PI), and 0.5% Oil Red O (ORO) solution in isopropanol were purchased from Sigma-Aldrich. RPMI-1640 with 2.05 mM L-glutamine was purchased from Lonza. Paraformaldehyde (PFA, 4%) in PBS was obtained from Santa Cruz Biotechnology. Calcein acetoxymethyl ester (Calcein AM), DiI, and dioctadecyloxacarbocyanine (perchlorate) (DiO) were purchased from Cayman Chemical. Copper sulfate and Simvastatin were purchased from AmBeed. RAW 264.7 NO(-) murine macrophage was a gift from Dr. Yong Wang’s laboratory at the Department of Biomedical Engineering, the Pennsylvania State University. Porcine hearts were obtained from a local slaughterhouse.

**Peptide Synthesis:** Peptide emulsifiers were synthesized by standard solid-phase peptide synthesis using a CEM Liberty Blue microwave peptide synthesizer with DIC/oxyma activation. Fmoc groups were removed by 20% piperidine in DMF. Crude peptide on resin was washed with DMF followed by DCM. Resin cleavage and side chain deprotection were achieved simultaneously by treating the resin with a cleavage cocktail (TFA/TIPS/distilled water = 95:2.5:2.5, v/v/v) for 2 hours under argon. Subsequently, the cleavage cocktail was mostly evaporated using argon, and then cold diethyl ether added to precipitate the crude peptide, which was dried by lyophilization. Purification was performed using reverse phase HPLC (Shimadzu) with Phenomenex semi-prep Luna C18 column at 17 °C using a gradient of 0−55% standard solvent B (90% acetonitrile, 9.9% water, 0.1% TFA) for 20 min, followed by 55−100% standard solvent B for 60 minutes. Standard solvent A was 0.1% TFA in water. The purified peptide was identified by LC-MS (Shimadzu, LCMS 2020) equipped with a Phenomenex C18 analytical column at 20 °C using a gradient of 1% standard solvent B (90% acetonitrile, 9.9% water, 0.1% FA) per minute for 100 minutes. 0.1% FA in water was used as standard solvent A for LC-MS.

**Emulsion Preparation:** 200 μL of PFP or PFH was added to a vial containing lyophilized peptide (~0.56 mg) and then vortexed, followed by sonication for 5 seconds to create a uniform dispersion. Subsequently, 3 mL of distilled water was added to the mixture followed by immediate ultrasonication for 3 minutes in sonic bath operating at 40 kHz and 120 W ([peptide] = 0.05 mM). Formation of the emulsions was confirmed using an inverted phase contrast microscope (Olympus IX50, Japan). PFH was employed for oxLDL-binding and *in vitro*, *ex vivo* plaque-targeting studies, while PFP was used otherwise. Emulsion concentration in the following studies was determined by flow cytometry-based particle counting.

For *in vitro* oxLDL-binding studies and time-dependent persistence assay in foam cells, DiO-loaded emulsions were prepared with the same procedure as described above, with the exception that 10 μL of 3 mM DiO in HFIP was mixed with the lyophilized peptide before PFH was added. For *ex vivo* plaque-targeting experiments, DiI-loaded emulsions were prepared using 2 mM DiI in HFIP. After emulsification, the dye-loaded emulsion solution was transferred into Slide-A-Lyzer dialysis cassettes (3.5kDa MWCO) and dialyzed against 2.5% DMSO solution overnight to remove unloaded dye and HFIP. Prior to retrieval of the dye-loaded emulsions, samples were dialyzed against distilled water for an hour to replace the residual DMSO with water. Dye-loading into the emulsions was confirmed using a microplate reader (Biotek Cytation 3) with a green fluorescent protein (GFP) filter cube (469/525 nm, Ex/Em) and Texas red filter cube (586/647 nm, Ex/Em) for DiO and DiI, respectively.

**Dynamic Light Scattering (DLS):** The size of the emulsion droplets was measured using Zetasizer Nano ZS (Malvern Panalytical, UK) at a scattering angle of 173° at 25 °C using a He-Ne laser operating at 633 nm. The emulsions were 10-fold diluted with distilled water to a final volume of 1 mL, which was then transferred into a polystyrene cuvette for the analysis. Data were collected in triplicate and are represented as a Z-average.

**Transmission Electron Microscopy (TEM):** The emulsion solution (2–3 μL) was deposited onto a formvar/carbon-coated copper grid (Ted Pella, Inc., Carbon Type-B, 200 mesh), and the solvent evaporated under ambient conditions. TEM imaging was conducted using a FEI Titan Tecni G2 microscope operating at an acceleration voltage of 200 kV.

For TEM analysis of oxLDL-treated RAW 264.7 cells incubated with or without the emulsions, cells were seeded in a 6-well plate at a density of 4 x 10^5^ cells per well and allowed to adhere overnight. The media was then replaced with fresh media containing oxLDL (20 μg mL^-1^), and incubated with oxLDL for 8 hours. Subsequently, the oxLDL-treated cells were washed with media and incubated with emulsions diluted 10-fold with cell culture media (~6 x 10^3^ emulsions mL^-1^). After 15 hours of incubation, the cells were washed with PBS to remove residual emulsions and retrieved by trypsinization using 0.25% trypsin-EDTA for 5 minutes at 37 °C. The cells were spun down by centrifugation (300 *g* for 3 minutes) and the supernatant aspirated, followed by re-suspension of the cells in 200 μL of media containing BSA (20 wt%) as a cryoprotectant.

TEM specimens were prepared *via* high-pressure freezing with the following details: the cell suspension in 20 wt% BSA was vitrified using a high-pressure freezing equipment (Leica EM HPM100). Subsequently, freeze-substitution was performed using a quick freeze-substitution method.^[2]^ Briefly, the frozen samples were immersed in a freeze substitution cocktail containing 1% osmium tetroxide, 0.1% uranyl acetate, and 0.05% water in acetone. The samples were then gradually warmed to 4°C overnight with mixing, followed by washing with 100% acetone several times and then embedded in Epon-Araldite epoxy resin. Thin sections of samples (< 100 nm in thickness) were obtained using an ultramicrotome (UC6, Leica Microsystems GmbH, Vienna, Austria) and then collected on formvar/carbon-coated copper grids (Ted Pella, Inc., Carbon Type-B, 200 mesh). The thin sections were further stained with 2% uranyl acetate and Reynold’s lead citrate. TEM imaging of the cell specimens was performed using Talos F200C (Thermo Fisher Scientific) or FEI Titan Tecni G2 microscope operating at an acceleration voltage of 200 kV.

**Stability Study:** Freshly prepared Apo-NPep emulsions (~6 x 10^4^ emulsions mL^-1^) with a PFP core were divided into two glass vials and stored at 4 °C and 37 °C, respectively. At each time point, 200 μL of the samples was transferred into a quartz cuvette with a 1 cm pathlength, and the optical density at λ = 600 nm (OD_600_) was measured using an ultraviolet/visible spectrometer (Cary 60 UV-Vis, Agilent Technologies). The data were collected in triplicates and are presented as the mean ± standard deviation.

To track the change of Apo-NPep size over 10 days, the emulsion solution (~6 x 10^4^ emulsions mL^-1^) was stored in a glass vial at 4 °C between measurements, and DLS analysis was performed at each time point following the procedure described above.

**OxLDL-Binding Assay:** Prior to *in vitro* study, the oxLDL-binding affinity of the emulsions were explored by combining the emulsion (~5 x 10^4^ emulsions mL^-1^) with 10 μg mL^-1^ DiI-oxLDL solution at a 1:9 volume ratio. An aliquot of the mixture was transferred onto a glass slide and covered with a coverslip, sealing the edges with nail polish to prevent sample evaporation. Imaging was performed using a confocal laser scanning microscope (Olympus Fluoview 1000) and the data was analyzed using ImageJ software.

For *in vitro* fluorescence microscopy, RAW 264.7 cells were seeded into a four-well chamber slide at a density of 8.6 x 10^4^ cells per well and cultured overnight. After 4-hour incubation with 5 μg mL^-1^ DiI-oxLDL, the cells were washed with media following treatment of DiO-loaded emulsions diluted by 10-fold with cell culture media (~5 x 10^3^ emulsions mL^-1^). After 6 or 24 hours of incubation, the cells were washed with cold PBS three times and then fixed with 4% PFA for 15 minutes at room temperature. Subsequently, the cells were rinsed with PBS, and the culture chamber was removed. A drop of ProLong Diamond Antifade Mountant with DAPI was placed onto the glass slide and covered with a coverslip, and stored at 4 °C until imaging. Z-stack images were constructed from individual 2D micrographs using ImageJ software.

***Ex Vivo* Plaque-Targeting Study:** All mice used in this study were housed in accordance with the husbandry guidelines established by the Institutional Animal Care and Use Committee (IACUC) at Pennsylvania State University, University Park Campus, which specifically approved this study. Four-week-old apolipoprotein E-deficient (ApoE−/−) female mice were provided with free access to water and were fed a high-fat diet ad libitum (41% Kcal from fat, 40% Kcal from carbohydrate, 18% Kcal from protein, and 0.15% cholesterol; Research Diets, New Brunswick, NJ, USA). After an average of 16 weeks on this diet, the mice were euthanized by carbon dioxide inhalation. Following the exposure of the aorta, 10 mL of cold PBS was perfused through the left ventricle using a syringe. The aorta was then removed, and the surrounding connective tissues were dissected away under a stereomicroscope. The excised aortas were kept on ice throughout the experiment. DiI-loaded Apo-NPep emulsions were injected into the aortas in a consistent flow direction from the abdominal to the ascending aorta, using a syringe equipped with a 30-gauge needle. After injection, the aorta was incubated for 10 minutes before being perfused with water to remove any unbound residual emulsions. Fluorescence imaging was performed using a CRi Maestro in-vivo imaging system with a yellow filter cube (670/630-800, Ex/Em) with an exposure time of 4000 ms. Subsequently, the aorta was then stained with 0.3% ORO in 60% isopropanol for 15 minutes at room temperature. The 0.5% ORO in isopropanol was diluted with water at a 6:4 ratio and filtered using a 0.22 μm syringe filter before use. The ORO-stained aorta was then washed with 60% isopropanol followed by water before imaging. The aorta that was not perfused with the emulsions was used as a control.

**US-Activation:** Apo-NPep emulsions containing PFP core (~6 x 10^4^ emulsions mL^-1^) were transferred (150 μL) into a 96-well plate and equilibrated at 37 °C for 20 minutes before measuring the initial OD_600_ prior to US-exposure. Then, each well loaded with emulsions was insonated for 1 minute with 50% duty cycle at an intensity of 0.1, 0.5, 1, and 2 W cm^-2^ using a Nepagene Sonitron GTS with a plane wave transducer (1 MHz, 6 mm diameter). The change in OD_600_ before and after US treatment was measured using a microplate reader (Biotek Cytation 3) and represented as percent activated emulsions (%) using the following equation: 100 – {(OD_600_ after US treatment – OD_600_ of water) / (OD_600_ before US treatment – OD_600_ of water) x 100%}. Data were calculated as mean ± standard deviation from triplicates.

**Macrophage Viability:** RAW 264.7 murine macrophages were cultured in RPMI-1640 medium supplemented with 10% FBS and 2.05 mM L-glutamine at 37°C in humidified atmosphere of 5% CO_2_. Biocompatibility of the emulsions was assessed using MTT assay by seeding RAW 264.7 cells in a 96-well plate at a density of 9 x 10^3^ cells per well, and allowing cells to adhere overnight. Cells were then washed with complete media, followed by treatment with media containing 10-fold diluted emulsions (~5 x 10^3^ emulsions mL^-1^), and then incubated for 24 hours at 37 °C and 5% CO_2_. Cells with blank media and media containing 20% DMSO were used as negative and positive controls, respectively. Subsequently, the supernatant was removed and 100 μL of 0.5 mg mL^-1^ MTT in cell culture media was added to the cells. After 3 hours of incubation to allow conversion of MTT to the formazan product, the supernatant was replaced with 100 μL of DMSO, followed by further incubation for 15 minutes at 37 °C to dissolve the formazan crystals (*n* = 10).

For evaluating cellular viability after US treatment in the presence of emulsions, a similar procedure was followed. RAW 264.7 cells were seeded in a 12-well plate at a density of 1.5 x 10^5^ cells per well and then incubated with the emulsions (~5 x 10^3^ emulsions mL^-1^) for 15 hours. Cells without the emulsions and with 20% DMSO were used as negative and positive controls, respectively. After replacing with fresh media, US was applied to the cells with or without the emulsions for 1 minutes with 50% duty cycle at an intensity of 0.1, 0.5, 1, and 2 W cm^-2^ using a Nepagene Sonitron GTS with a plane wave transducer (1 MHz, 20 mm diameter). For all MTT assays, absorbance at λ = 540 nm was recorded using a plate reader (Biotek Cytation 3), and the cell viability (%) was calculated using the following equation: (λ_treated_ – λ_positive control_) / (λ_negative control_ – λ_positive control_) x 100%. Data were calculated as mean ± standard deviation from triplicates.

For live/dead imaging assay on the RAW 264.7 cells with the emulsions, the same protocol used for MTT assay was conducted except that the media was replaced with 100 μL of Calcein AM solution (3 μM) instead of MTT solution and then incubated for 30 minutes at 37 °C. Thereafter, 15 μL of PI (1.5 mM) in growth media was added into each well and then incubated for another 30 minutes at 37 °C. The fluorescence imaging was performed using a microplate reader (Biotek Cytation 3) with GFP filter cube for Calcein and Texas Red filter cube for PI.

***In Vitro* oxLDL Efflux Assay:** To generate foamy macrophages, RAW 264.7 cells were seeded in 12-well plates at a density of 2.5 x 10^5^ cells per well and cultured overnight. Afterwards, the cell culture media was replaced with serum free RPMI-1640 media containing DiI-oxLDL (5 μg mL^-1^) and incubated for 4 h. Then, cells were washed with serum-free media and then treated with the emulsions, 10-fold diluted with serum free media (~5 x 10^3^ emulsions mL^-1^). The original emulsion solution was diluted with water by 25-fold before further dilution with the media (final [peptide] = 0.2 μM). At each time point, the cells were washed with cold PBS three times and retrieved by scraping them off the culture substrate. Cells were spun down by centrifugation (400 *g* for 5 minutes), followed by decanting the supernatant and then fixed with 0.1% PFA for 15 minutes at room temperature. Cells were centrifuged again (400 *g* for 5 minutes) and resuspended with PBS after decanting the supernatant. Flow cytometry was performed on Attune NxT Acoustic Focusing Cytometer with blue laser at 488 nm with emission filter 574/26 nm and analyzed using FlowJo 10.8 software (BD Biosciences, Franklin Lakes, NJ, USA) and the oxLDL efflux (%) was calculated using the following equation: 100 – {(Geom. MFI_emulsions+US_ – Geom. MFI_naïve cell without DiI-oxLDL_) / (Geom. MFI_untreated control_ – Geom. MFI_naïve cell without DiI-oxLDL_) x 100%}. Data were calculated as mean ± standard deviation from triplicates.

For US-treatment studies, RAW 264.7 cells were seeded in 12-well plates at a density of 1 x 10^5^ cells per well and cultured overnight. The DiI-oxLDL-treated RAW 264.7 cells were prepared using the same protocols described above. After incubation with the emulsions (~6 x 10^3^ emulsions mL^-1^) for 24 h, cells were washed and replenished with fresh serum-free media (1.5 mL) and US was applied at different intensities (0, 0.1, 0.5 and 1 W cm^-2^, 1 MHz, 50% duty cycle, 1 min. exposure; for comparison study of Apo-NPep with Scr-NPep, 0.5 W cm^-2^ was used.) using a Nepagene Sonitron GTS with a plane wave transducer (20 mm diameter) at room temperature. Following a 1-hour incubation at 37 °C, the cells were collected for flow cytometry with the same protocols described above.

For the efflux study of Simvastatin-loaded Apo-NPep emulsions by insonation, the samples were prepared as follows: 50 μL of 65 mM Simvastatin in ethanol was added to a glass vial containing lyophilized peptide powder (~0.56 mg), followed by addition of 200 μL of PFP into the vial. After vortexing and sonicating the mixture for approximately 10 seconds, 3 mL of water was then added into the vial, following immediate ultrasonication for 3 minutes. The Simvastatin-loaded emulsions were dialyzed against water for 24 hours to remove unloaded drugs. The retrieved Simvastatin-loaded emulsions were diluted 10-fold with water and further diluted with serum-free media by 10-fold before 24 hours of incubation with the DiI-oxLDL-treated RAW 264.7 cells ([peptide] = 0.5 μM). Ultrasonic application and subsequent flow cytometry were conducted using the same protocol as mentioned above.

**Circular Dichroism (CD) Spectroscopy:** CD spectra were collected on a Jasco J1500 CD spectrometer at 25 °C using a 0.1 cm path length quartz cuvette with a bandwidth of 1 nm and integration time of 4 seconds. Spectra were recorded from 190 nm to 260 nm with a scanning speed at 50 nm min^-1^ and scans were repeated three times and averaged.

***In Vitro* US Imaging:** The tissue-mimicking agar phantom was prepared by molding 1.7 mL centrifuge tubes in 1.5% agar gel (w/v) within a bottomless 24-well plate sealed with a transparent film dressing (Tegaderm^TM^, 3M). After loading the samples (200 μL of Apo-NPep emulsion, Apo-NPep-foam cells, or normal foam cells) diluted 5-fold with degassed PBS, the top of the well plate was sealed with a film dressing to submerge the plate into the water tank. Any unloaded wells or empty spaces within the plate were filled with degassed PBS before complete sealing.

Prior to *in vitro* US imaging, Apo-NPep emulsions were imaged within agar phantoms using L22-14v (18.5 MHz) or L7-4 (5.2 MHz) transducers. The well plate was placed on a neoprene block and submerged in a degassed water tank heated to 37 °C. Imaging was performed after allowing the plate to equilibrate at 37 °C for 15 minutes inside the tank. The US imaging capabilities of the emulsions were evaluated using a Vantage-128 research ultrasound system (Verasonics, Kirkland, WA, USA) with the following conditions for B-mode using L22-14v transducer: 18.5 MHz, up to peak pressures of 1.0 MPa positive and 0.6 MPa negative, 1-cycle pulses repeated at 3000 Hz. The mean pixel intensity of the region of interest (ROI) was quantified using ImageJ software, maintaining consistent ROI dimensions across all experiments. For color Doppler US superimposed with B-mode imaging, L7-4 transducer was used with the following conditions: 5.2 MHz, peak pressure of 2.6 MPa positive and 0.9 MPa negative for B-mode and peak pressure up to 3.8 MPa positive and 1.6 MPa negative for color Doppler using 7 ensembles comprised of 12 cycles, repeated at 3000 Hz. For quantification of the Doppler signal, data were saved at discrete time points.

For B-mode imaging of Apo-NPep as a function of emulsion concentration (~2 x 10^3^ – 6 x 10^3^ emulsions per agar cavity) and multi-day US persistence studies, a portable L6C US transducer (SonoQue, CA, USA) was used with the following conditions: 7.5 MHz, mechanical index: 0.7. The mean pixel intensity of the ROI was quantified using ImageJ software, maintaining consistent ROI dimensions across all experiments.

*In vitro* B-mode and Doppler imaging of Apo-NPep-foam cells in agar phantoms were performed by seeding RAW 264.7 cells in a 6-well plate at a density of 2 x 10^5^ cells per well, followed by overnight culture. Subsequently, oxLDL (10 μg mL^-1^) was added to the cells to generate lipid-laden macrophages. After 4 hours of incubation, the cells were washed and treated with emulsions after 10-fold dilution with growth media (~5 x 10^3^ emulsions mL^-1^). The oxLDL treated RAW 264.7 cells were used as a control. Following 14 hours of incubation, the cells were washed with PBS, collected by scraping them off. Excess PBS was removed by spinning down the Apo-NPep-foam cells at 300 *g* for 3 minutes. The cells were stored on ice and shortly used for US imaging studies after re-dispersing them in degassed PBS by 5-fold dilution. The same acoustic conditions employed for the emulsion were used for *in vitro* imaging. A quantitative analysis was conducted to compare the contrast of Doppler twinkling and B-mode imaging by extracting a signal-to-noise ratio from the data using ImageJ. Specifically, in B-mode images, the mean pixel intensity was measured within a defined ROI containing the samples and compared to a background region of the same area size. For Doppler US, the twinkling signal was delineated using color thresholding in ImageJ and compared to the surrounding background.

For long-term persistence Doppler imaging, RAW 264.7 cells were seeded in a 12-well plate at a density of 2.5 x 10^3^ cells per well and allowed to adhere overnight. At each time point, the cells were treated with Apo-NPep emulsions after 10-fold dilution with the cell culture media (~1.5 x 10^4^ emulsions mL^-1^), maintaining a similar concentration of emulsions by measuring their optical density before treatment. RAW 264.7 cells untreated with Apo-NPep emulsions were used as a control. Prior to retrieval, the cells were imaged with an inverted phase contrast microscope (Olympus IX50, Japan) (Figure S21), and then collected by scraping them off of the plate and centrifugation at 400 *g* for 5 minutes. After decanting the culture media, the cells were resuspended with degassed PBS (5x dilution) and then loaded into agar cavities (~1.7 x 10^5^ cells per cavity) to be imaged using a portable L5C US transducer (SonoQue, CA, USA) with the following conditions for color Doppler: 6.5 MHz, peak pressure of 2.2 MPa positive and 1.8 MPa negative and for B-mode: 7.5 MHz, mechanical index: 0.7.

***Ex Vivo* US Imaging:** Apo-NPep emulsions and the Apo-NPep-foam cells were prepared using the same protocol for *in vitro* US imaging described above, with a cell density of 3.5 x 10^5^ cells per well in a 6 well-plate. After 5-fold dilution with degassed PBS, the samples were equilibrated at 37 °C for 10 minutes and then manually injected beneath the vasculature of a porcine heart using a 1 mL syringe equipped with an 18-gauge needle. The porcine heart was placed on a neoprene block in a water tank filled with degassed water at room temperature. The color Doppler US images were superimposed with B-mode using an L7-4 transducer (5.2 MHz, peak pressure of 2.6 MPa positive and 0.9 MPa negative for B-mode, peak pressure of 4.0 MPa positive and 1.8 MPa negative for Doppler using 7 ensembles comprised of 12 cycle pulses repeated at 3000 Hz). Degassed PBS and the oxLDL-treated RAW 264.7 cells (non-labeled foam cells) without internalized emulsions were used as controls for the emulsions and Apo-NPep-foam cells, respectively. For the quantification of the color Doppler signals, in-phase/quadrature (1/Q) Doppler data was saved at discrete time points.

**Passive Cavitation Detection:** To monitor the cavitation activity of the emulsions a passive cavitation detection (PCD) setting was employed using 1.07 MHz focused ultrasound (fUS) transducer (64-mm diameter and 52-mm focal length) with an *f*-number of 0.7 (H-102, Sonic Concepts, Bothell, WA, USA) and a passive receiving transducer (Y-107, Sonic Concepts, Bothell, WA, USA). fUS was triggered by a waveform generator (33600A Series, Keysight, Santa Rosa, CA, USA) (number of waveforms: 100) with 10 msec. intervals for 2 minutes (50% duty cycle, 50 Hz pulse repetition frequency, and 20 msec. of burst period), amplified by a linear radiofrequency power amplifier (55 dB, A500, Electronic Navigation Industries, Rochester, NY, USA), and transmitted to the fUS transducer through a matching network (Sonic Concepts, Bothell, WA, USA). The agar phantom (60 mm x 42 mm x 40 mm) was prepared as a cubic mold with a 5 mL of cavity, which was loaded with the emulsions (2 mL), 5-fold diluted with degassed PBS. Subsequently, the phantom was partially submerged in a degassed water tank heated to 37 °C. Degassed PBS (2 mL) was used as a control. The center of the samples within a cavity was positioned within the focal area of the acoustic field, which was adjusted using a L7-4 transducer (5.2 MHz), positioned at an angle of 90° to the fUS. The received US signals were processed using MATLAB (R2011a, The MathWorks, Natick, MA, USA).

**Statistical Analysis:** Grouped data are shown as mean ± standard deviation, with no additional filtering or removal of outliers performed. Statistical significance was assessed using GraphPad Prism 9.3.1 software. Unpaired two-sided *t*-test was performed to assess the statistically differences between two groups. For multiple comparisons, one-way ANOVA was performed. Relevant *p* values are indicated in the accompanying figure caption where appropriate.

| **US activation and oxLDL efflux study** | | |
| --- | --- | --- |
| Frequency | 1 MHz | |
| Duty cycle | 50% | |
| Exposure time | 1 min. | |
| Acoustic intensity | 0 ‒ 2 W cm^-2^ | |
|  | | |
| **B-mode US** | | |
| *In vitro* (Figure 4a ‒ d, Figure S19) | | *Ex vivo* (Figure 4f ‒ h) |
| Frequency | 18.5 MHz | 5.2 MHz |
| Acoustic pressure | p+ = 0.1 MPa, p-= 0.1 MPa or  p+ = 1.0 MPa, p- = 0.6 MPa | p+ = 1.9 MPa, p- = 0.7 MPa |
|  | | |
| **Doppler US** | | |
| *In vitro* (Figure 5a, Figure S20) | | *Ex vivo* (Figure 5b ‒ i) |
| Frequency | 5.2 MHz | 5.2 MHz |
| Acoustic pressure | For Doppler | |
|  | p+ = 3.8 MPa, p- = 1.6 MPa | p+ = 4.0 MPa, p- = 1.8 MPa |
|  | For B-mode | |
|  | p+ = 2.6 MPa, p- = 0.9 MPa | p+ = 2.6 MPa, p- = 0.9 MPa |
| Color priority | 180 | 255 |
| Color persistence | 81 | 0 |
| Doppler power threshold | 0.26 | 0.20 |
|  | | |
| **Persistence of twinkling** | | |
| Frequency | 6.5 MHz (for B-mode; 7.5 MHz) | |
| Acoustic pressure | p+ = 2.2 MPa, p- = 1.8 MPa | |
| Mechanical index for B-mode | 0.7 | |
|  |  | |
| **Passive cavitation detection (PCD)** | | |
| Frequency | 1.07 MHz (for B-mode; 5.2 MHz) | |
| Acoustic pressure | p+ = 1.3 MPa, p- = 1.0 MPa or  p+ = 2.5 MPa, p- = 2.0 MPa | |

# **Table S1.** US parameters employed.

#

# **
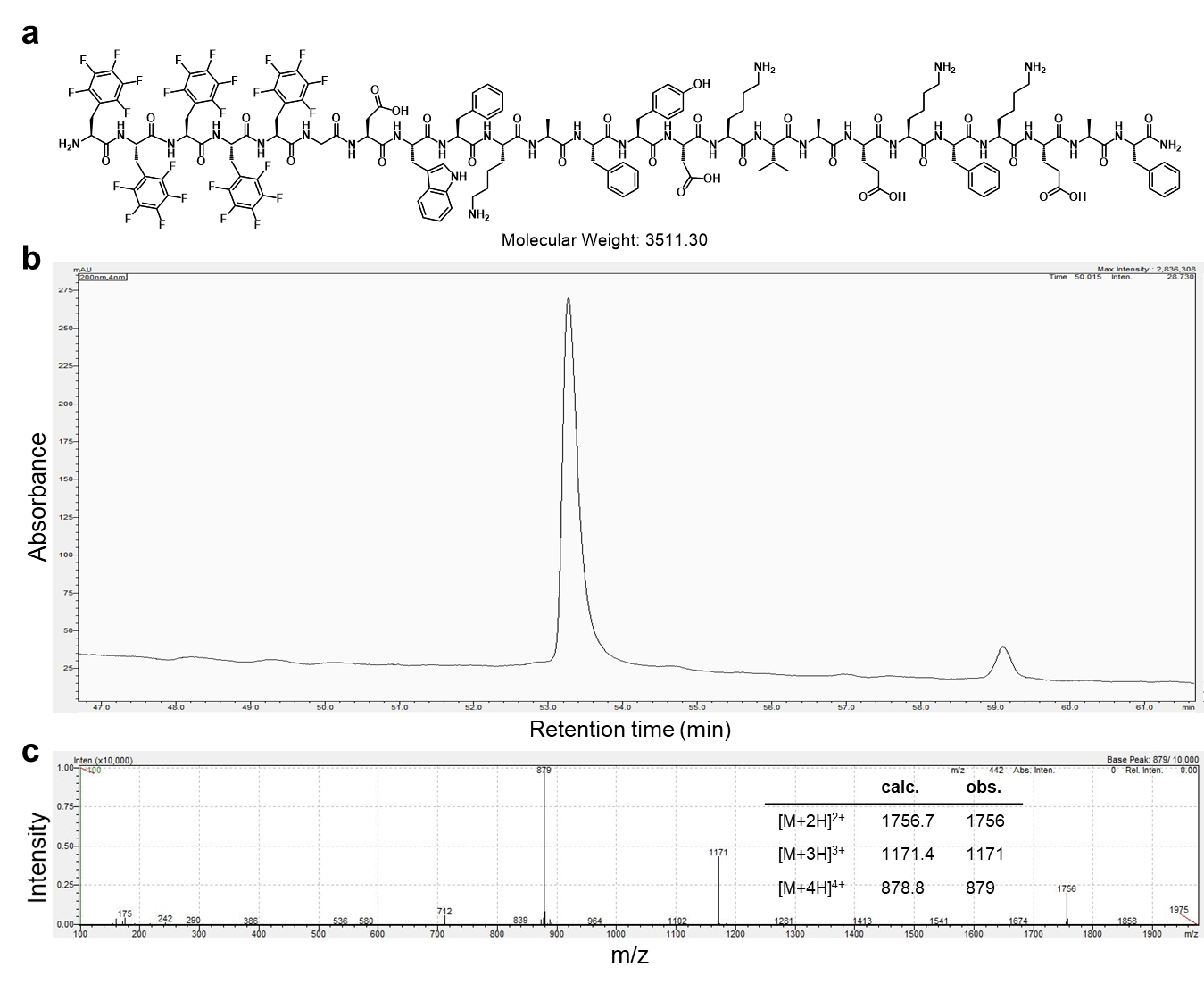
**

# **Figure S1.** a) Molecular structure of Apo-peptide emulsifier (H_2_N- F_F_F_F_F_F_F_F_F_F_GDWFKAFYDKVAEKFKEAF-NH_2_) and its b) HPLC trace and c) mass spectrum.

# **Figure S2.** Time-dependent change of Apo-NPep size during storage at 4 °C. One-way ANOVA was used to determine statistical significance relative to Day 0; *n* = 7, ns: not significant.

**
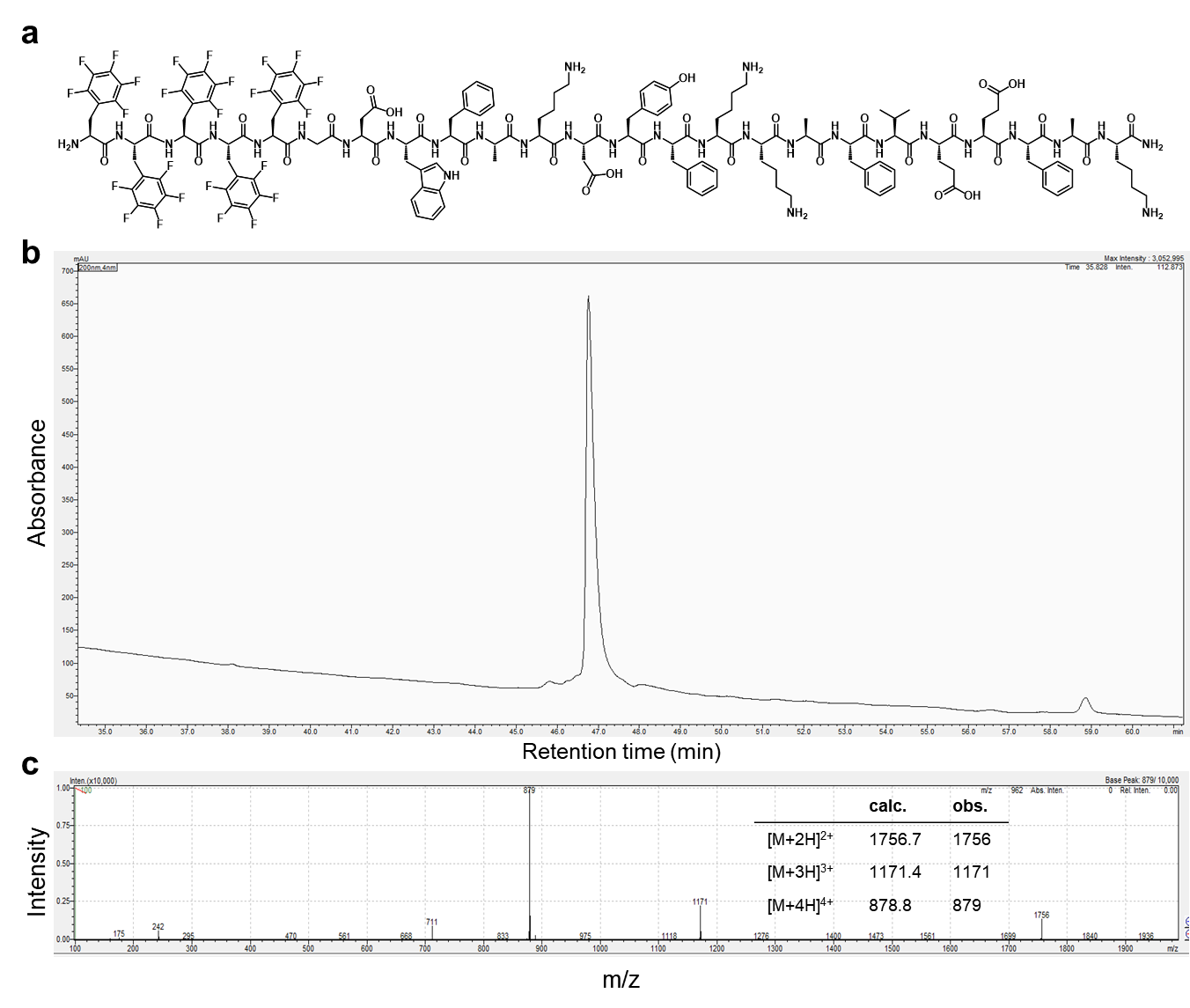
**

# **Figure S3.** a) Molecular structure of Scr-peptide emulsifier (F_F_F_F_F_F_F_F_F_F_GDWFAKDYFKKAFVEEFAK-NH_2_) and its b) HPLC trace and c) mass spectrum.

# **Figure S4.** Viability of RAW 264.7 cells after 24 hours of incubation with the indicated emulsions. Statistical analysis was performed *via* unpaired *t*-test compared to nontreated control; *n* = 10, ns: not significant.


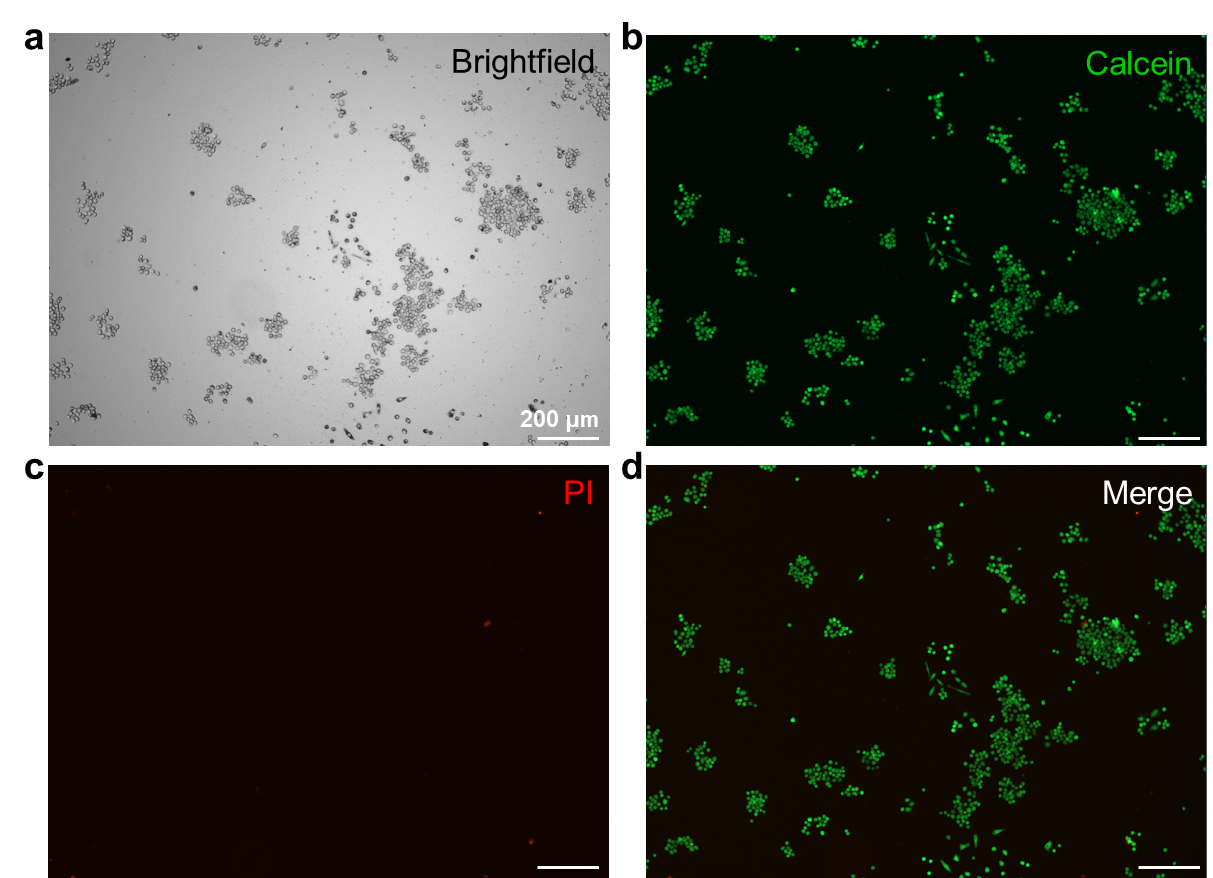


# **Figure S5.** Optical and fluorescence micrographs of live/dead assay using RAW 264.7 cells treated with Apo-NPep emulsions for 24 hours; a) brightfield, b) green fluorescence from live cells stained with Calcein, c) red fluorescence from dead cells stained with PI, and d) merged image of (b,c).


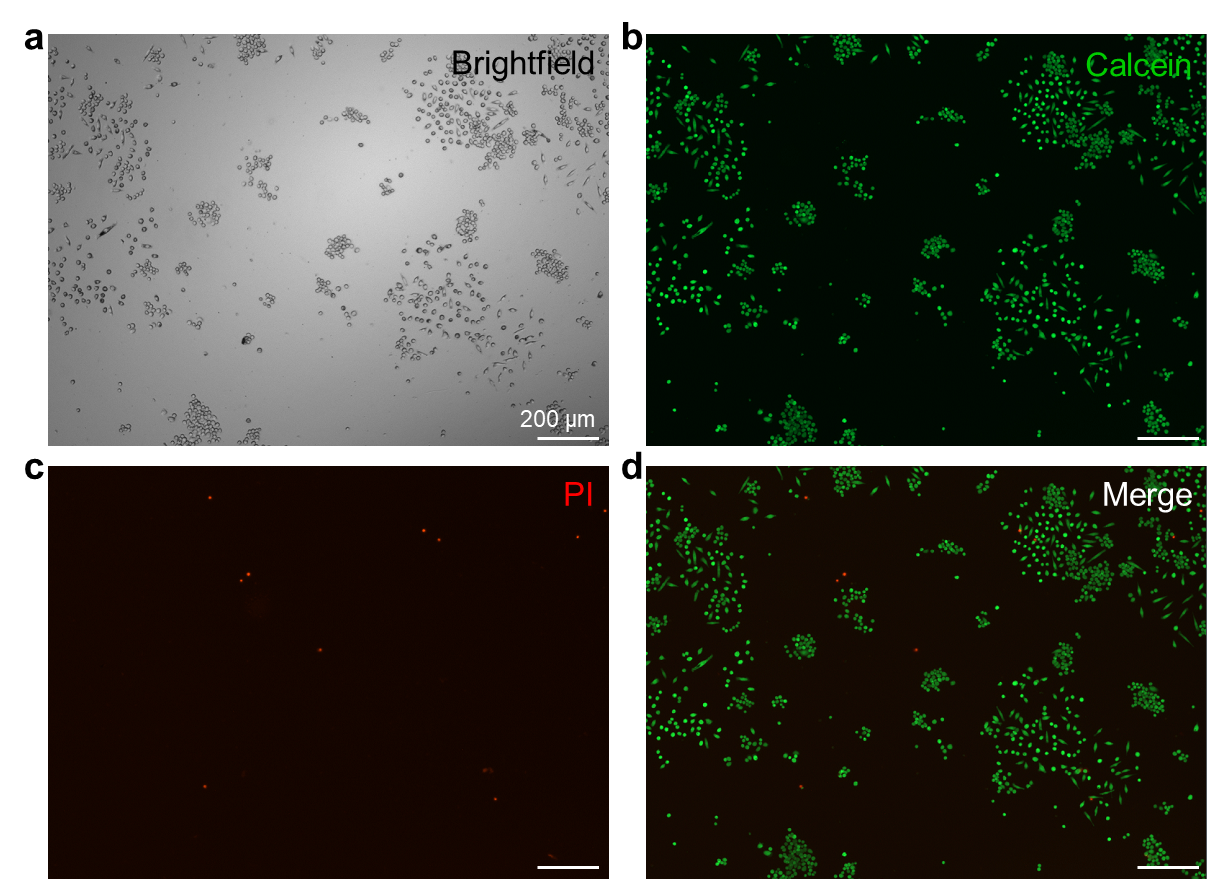


# **Figure S6.** Optical and fluorescence micrographs of live/dead assay using RAW 264.7 cells treated with Scr-NPep emulsions for 24 hours; a) brightfield, b) green fluorescence from live cells stained with Calcein, c) red fluorescence from dead cells stained with PI, and d) merged image of (b,c).


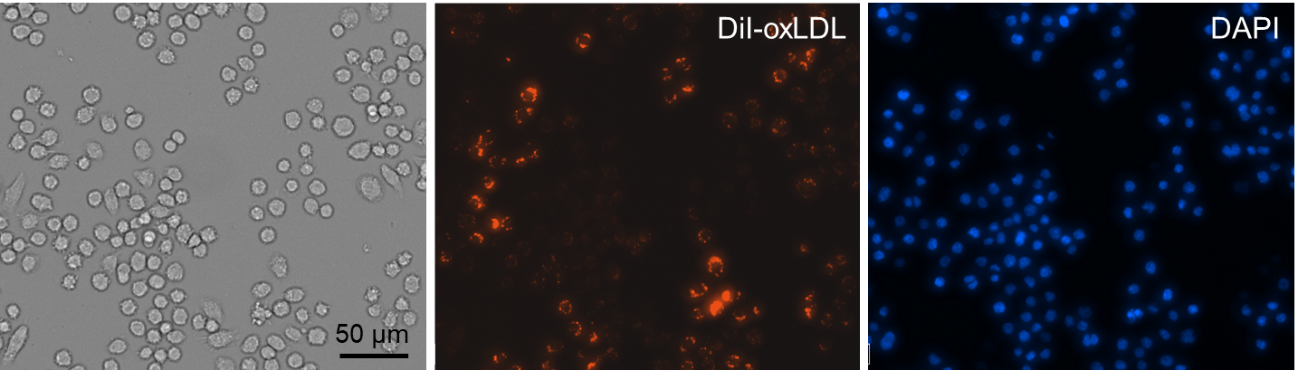


# **Figure S7.** Epifluorescence micrographs of RAW 264.7 cells incubated with DiI-oxLDL (10 μg mL^-1^) for 4 hours. Red fluorescence: DiI-oxLDL and blue fluorescence: DAPI from nuclei.


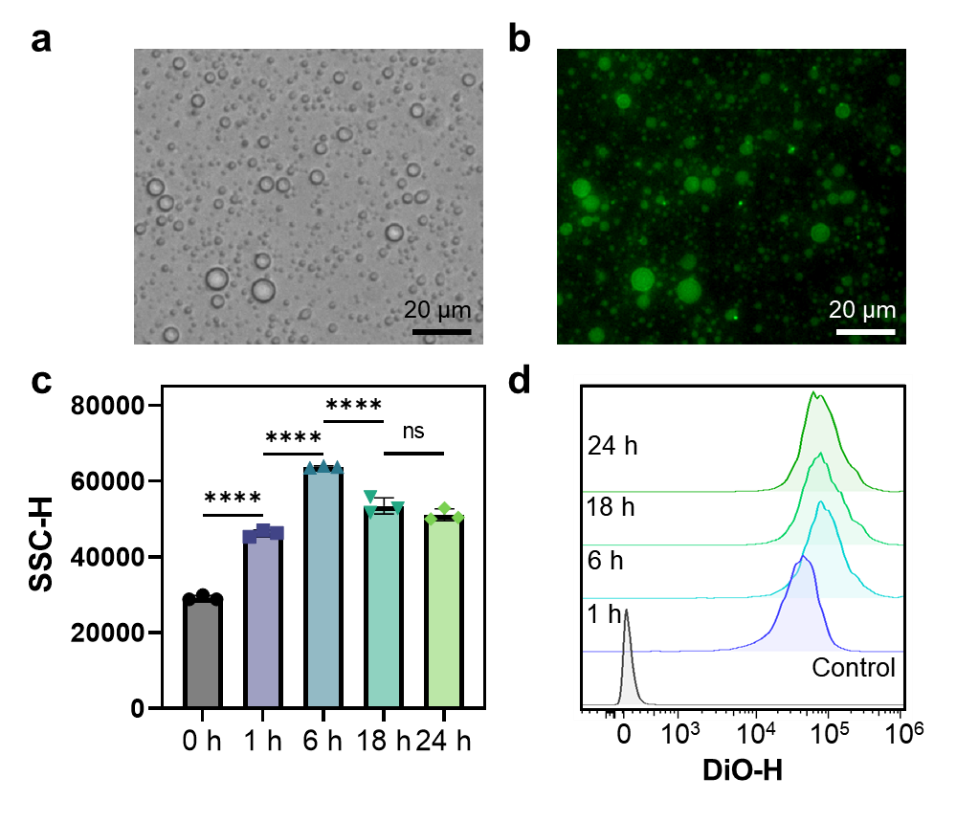


# **Figure S8.** Time-dependent internalization of DiO-loaded Apo-NPep emulsions into oxLDL-treated RAW 264.7 cells. a) Brightfield and b) fluorescence images of DiO-loaded emulsions. c) Side scattering intensity from the oxLDL-treated RAW 264.7 cells incubated with DiO-loaded emulsions at the indicated incubation time. Statistical analysis was performed *via* one-way ANOVA; *n* = 3, *****p* < 0.0001, ns: not significant. d) Fluorescence histogram of DiO-loaded emulsions within the oxLDL-treated RAW 264.7 cells analyzed by flow cytometry at the indicated time point. Control is non-treated foamy macrophages.


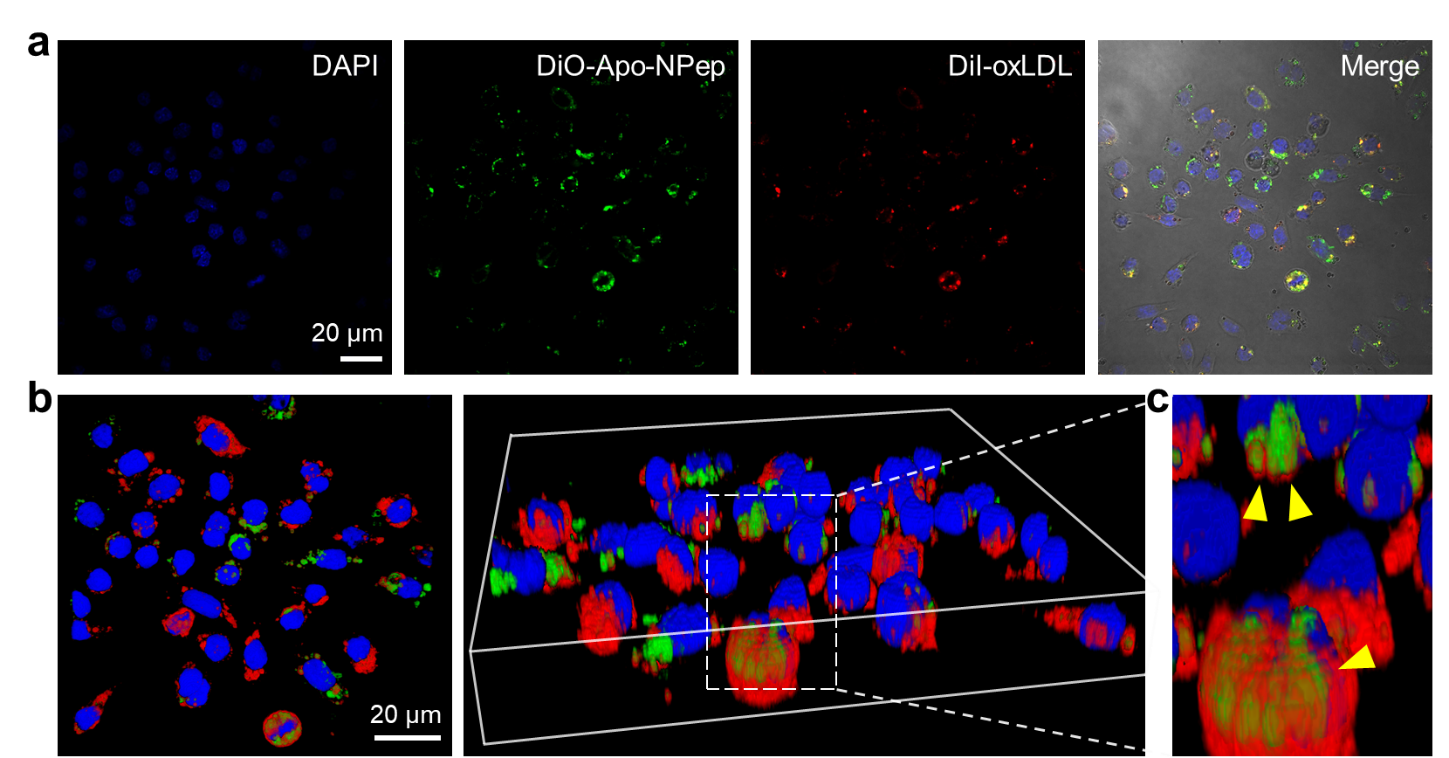


# **Figure S9.** a) Confocal laser scanning micrographs of RAW 264.7 cells treated with DiI-oxLDL for 4 hours followed by incubation with DiO-loaded Apo-NPep emulsions for 24 hours. b) Reconstructed orthographic fluorescent micrographs showing co-localization of DiO-loaded emulsions (green) and DiI-oxLDL (red) in macrophages. c) Magnified Z-stack image shows the interaction between DiI-oxLDL and DiO-loaded Apo-NPep emulsions, highlighted by yellow triangles.


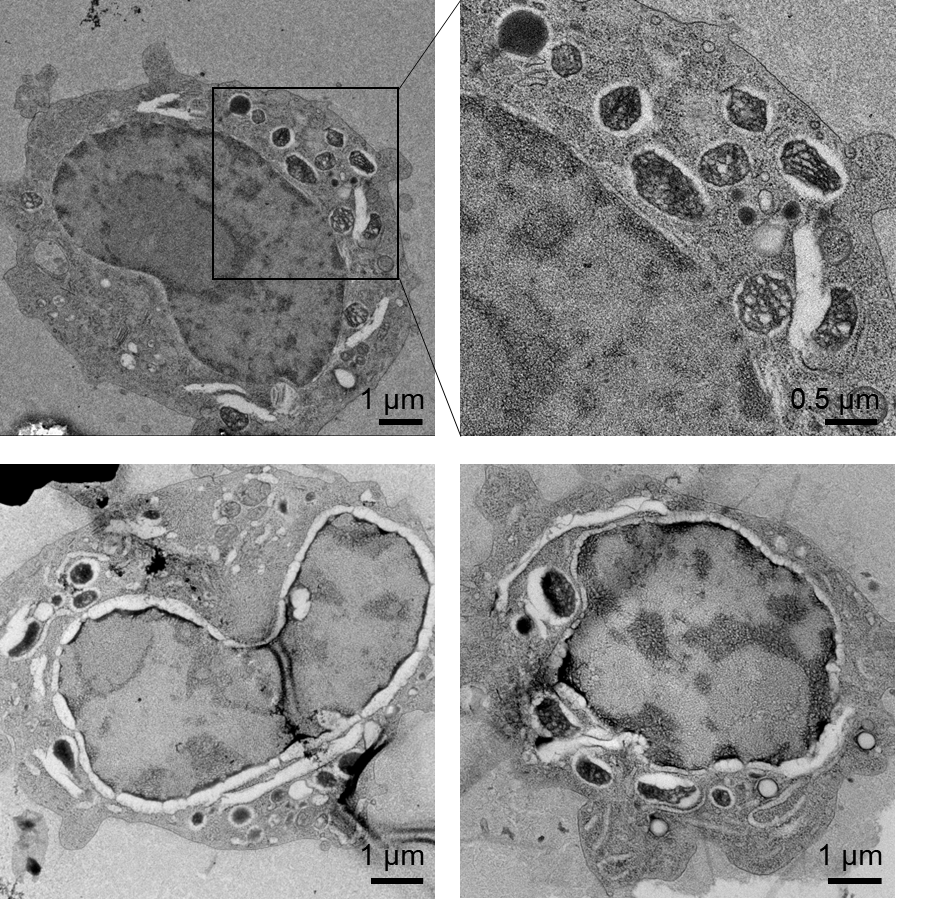


# **Figure S10.** Replicate TEM images of oxLDL-treated RAW 264.7 cells following 15 hours of incubation with Apo-NPep emulsions.

# **
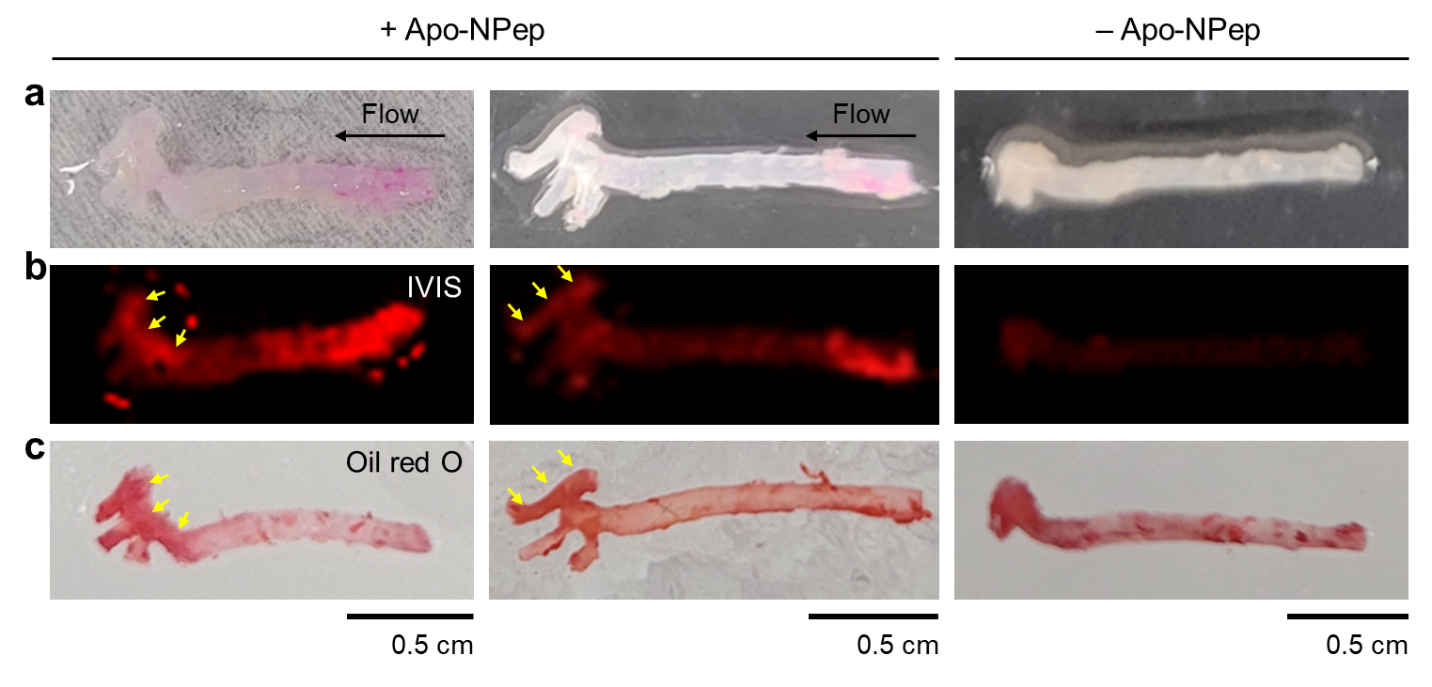
Figure S11.** a) Photographs and b) IVIS images of aorta obtained from high-fat diet-fed ApoE‒/‒ mice after perfusion with (+Apo-NPep) or without DiI-loaded Apo-NPep emulsions (-Apo-NPep). c) Photographs of each aorta after staining with ORO for 15 minutes to label atherosclerotic lesions. Yellow arrows highlight anatomical co-localization of Apo-NPep and ORO signals.


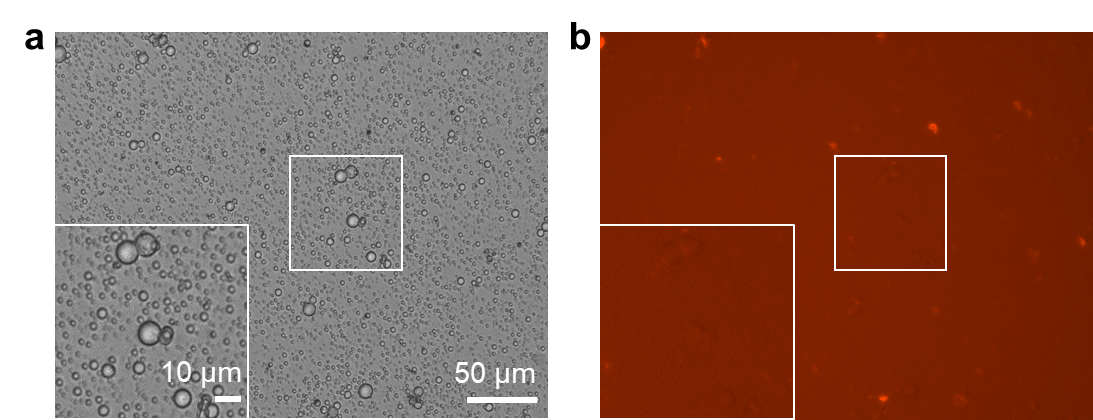


# **Figure S12.** a) Brightfield and b) red fluorescence micrographs (Texas red filter cube; excitation/emission: 586/647 nm) of the Apo-NPep emulsions incubated with ORO in 60% isopropanol solution for 20 minutes. Magnified images demonstrate that ORO does not interact with the emulsions.


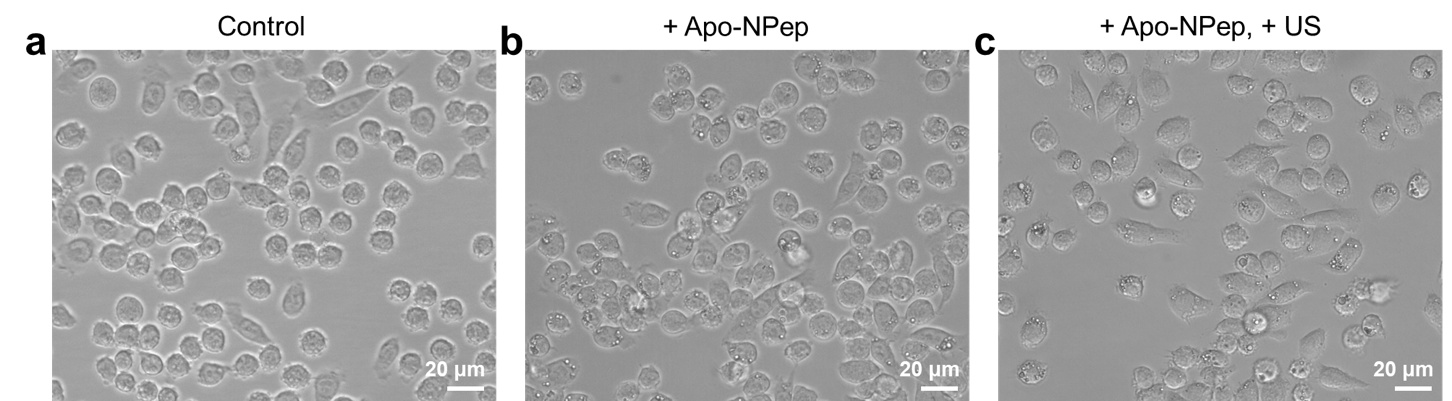


# **Figure S13.** Optical images of RAW 264.7 cells; a) non-treated control, b) cells after 15 hours of incubation with Apo-NPep emulsions, and c) emulsion-loaded cells following US treatment (1 MHz, 2 W cm^-2^, 50% duty cycle, 1 min. exposure).


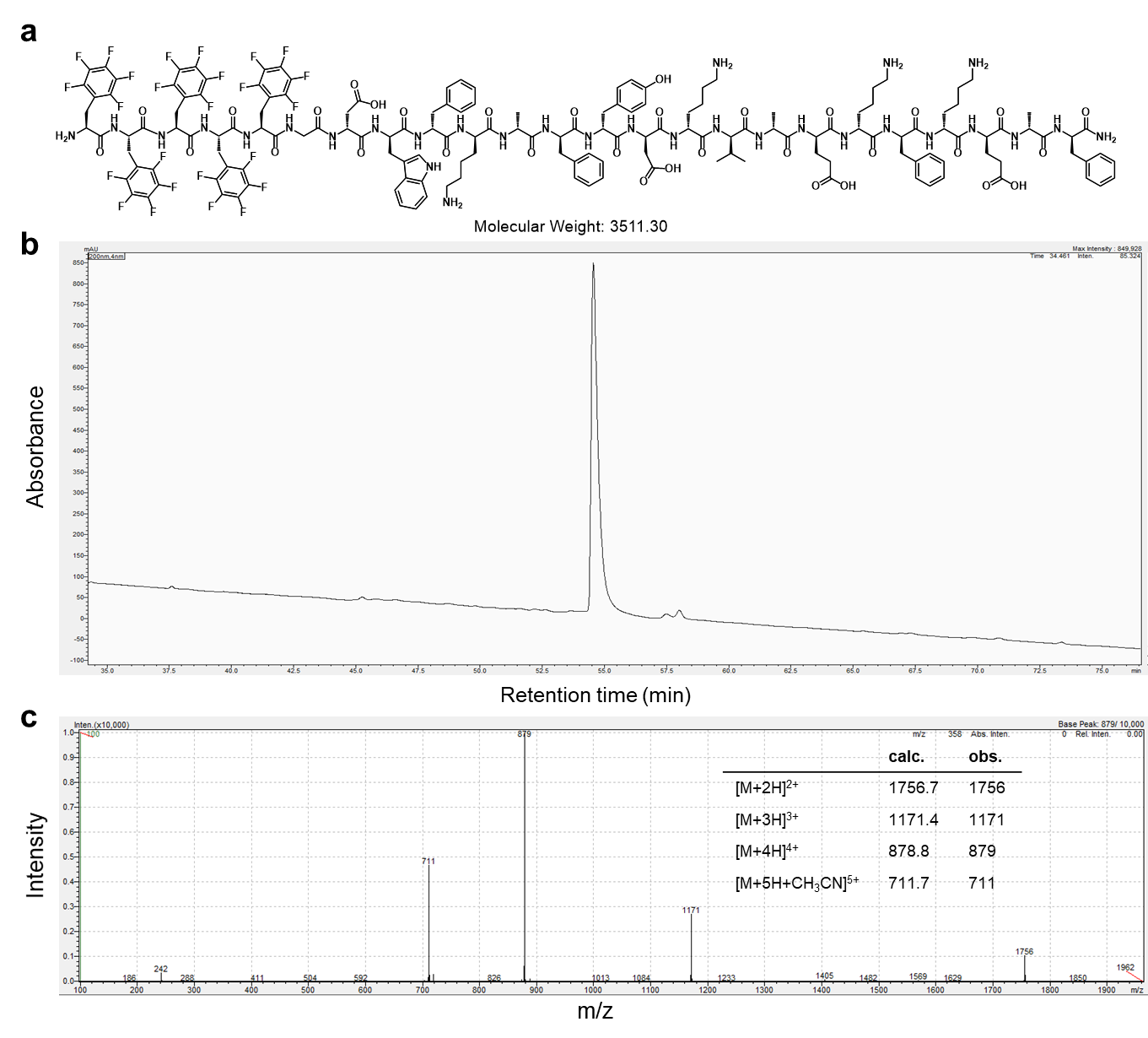


# **Figure S14.** a) Molecular structure of D-Apo peptide emulsifier (H_2_N- F_F_F_F_F_F_F_F_F_F_GDWFKAFYDKVAEKFKEAF-NH_2_; D-form amino acids are underlined) and its b) HPLC trace and c) mass spectrum.


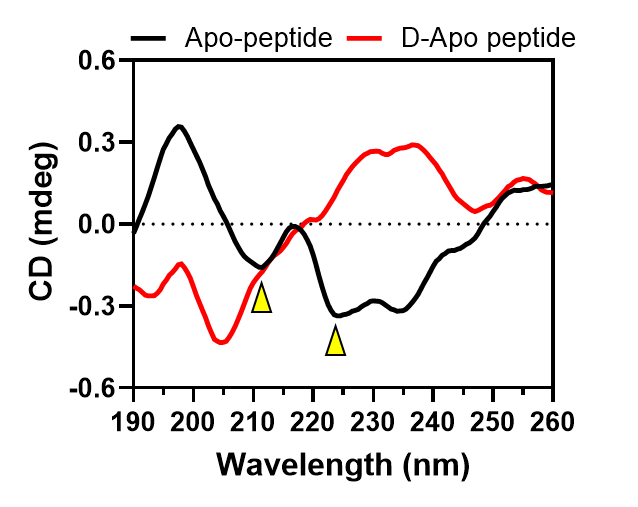


# **Figure S15.** CD spectra of the Apo- and D-Apo peptide emulsifiers in water. The yellow triangles at 211 and 223 nm from Apo-peptide emulsifier (black) highlight canonical α-helical features.

# **Figure S16.** Percent oxLDL efflux from RAW 264.7 foam cells in the absence (control) or presence of internalized Scr-NPep or Apo-NPep emulsions activated by US (1 MHz, 0.5 W cm^-2^, 50% duty cycle, *n* = 3).

# **Figure S17.** Relative increase in B-mode US signal as a function of Apo-NPep concentration (~2 x 10^3^ – 6 x 10^3^ emulsions per agar cavity). The B-mode signal intensity at the lowest emulsion concentration was normalized to 1, and the subsequent US intensities were presented as fold changes.


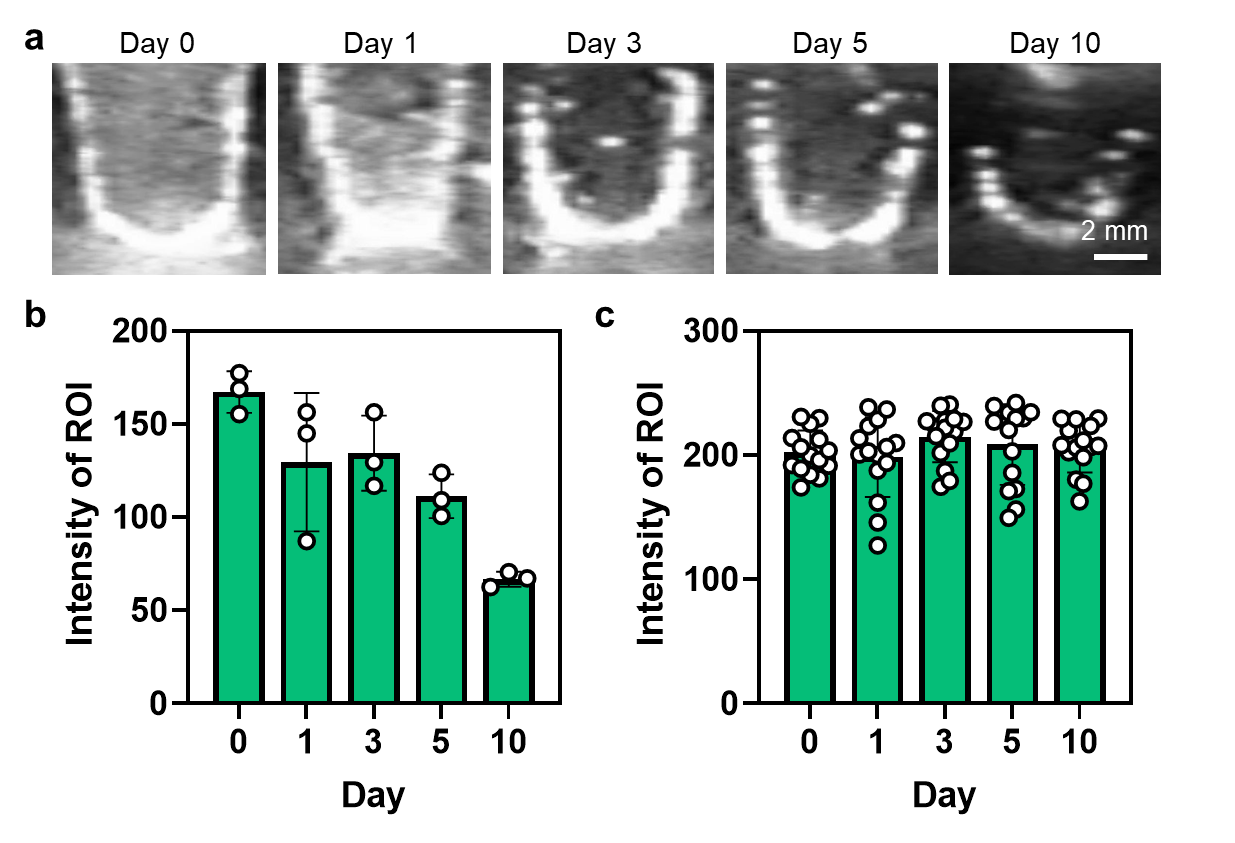


**Figure S18.** US imaging performance of Apo-NPep emulsions during 0 ‒ 10 days in 4 °C storage. a) Representative B-mode images of Apo-NPep emulsions (7.5 MHz, mechanical index: 0.7) at specified time points. b) Corresponding B-mode signal intensity from defined ROI of agar cavity in panel (a) (*n* = 3). c) Quantification of B-mode signal from individual emulsions measured at the indicated time point (*n* = 15).

#
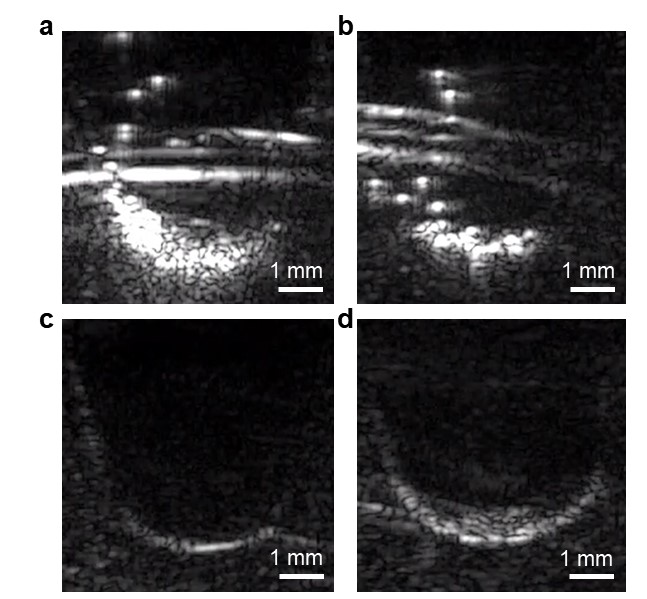
**Figure S19.** Representative B-mode images (18.5 MHz, p+ = 1.0 MPa, p- = 0.6 MPa) of oxLDL-treated RAW 264.7 cells a,b) with and c,d) without Apo-NPep emulsions.


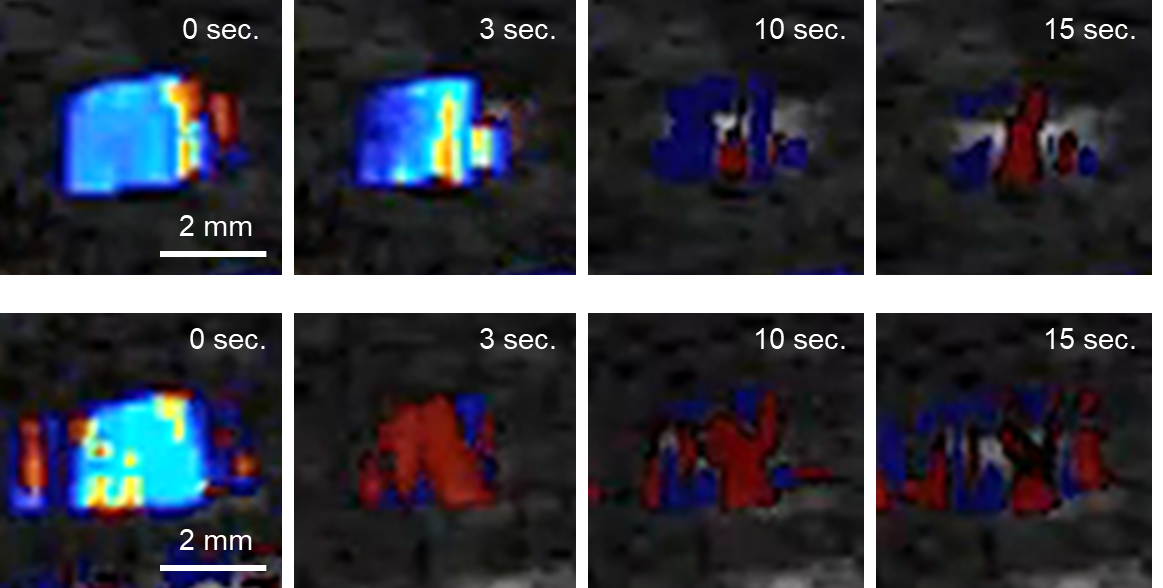


# **Figure S20.** Representative color Doppler US (5.2 MHz, p+ = 3.8 MPa, p- = 1.6 MPa) still images of an agar phantom loaded with Apo-NPep emulsions, superimposed on B-mode images (5.2 MHz, p+ = 2.6 MPa, p- = 0.9 MPa). Color priority: 180, color persistence: 81, and Doppler power threshold: 0.26.


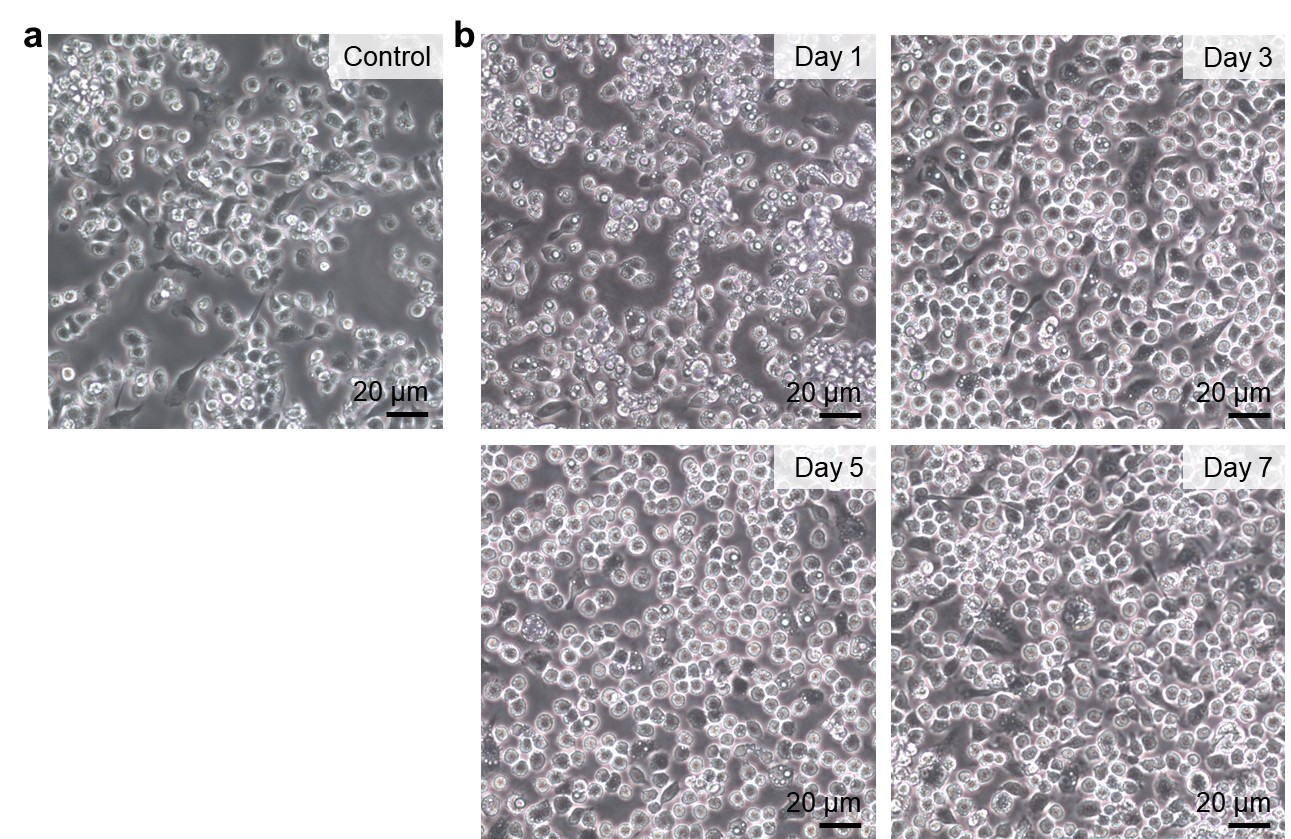


# **Figure S21.** Optical micrographs of RAW 264.7 cells a) without and b) with Apo-NPep emulsions incubated for 1 – 7 days.


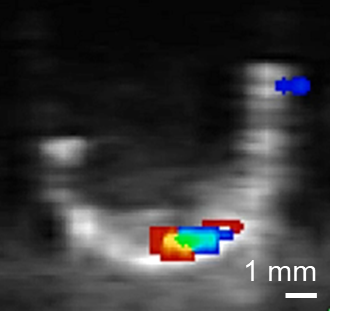


# **Figure S22.** Color Doppler US image of >2-month-old Apo-NPep emulsions superimposed on B-mode images (Doppler conditions: 6.5 MHz, p+ = 2.2 MPa, p- = 1.8 MPa, B-mode conditions: 7.5 MHz, mechanical index: 0.7).


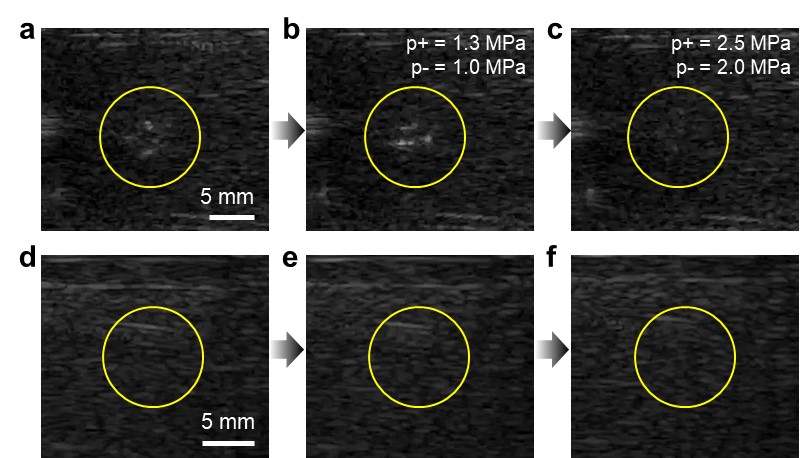


# **Figure S23.** B-mode still images (5.2 MHz, p+ = 0.1 MPa and p- = 0.1 MPa) of agar phantom loaded with a‒c) Apo-NPep and d‒f) degassed PBS a,d) before and b,c,e,f) after fUS-treatment b,e) at p+ = 1.3 MPa, p- = 1.0 MPa and c,f) at p+ = 2.5 MPa, p- = 2.0 MPa. Insonation with increased acoustic pressure (c,f) was followed after US-exposure with lower pressure (b,e). The yellow circles indicate the focal region in the agar cavity.

# **References**

[1] T.-W. Lian, L. Wang, Y.-H. Lo, I.-J. Huang, M.-J. Wu, *Biochim. Biophys. Acta* **2008**, *1781*, 601–609.

[2] K. L. McDonald, R. I. Webb, *J. Microsc.* **2011**, *243*, 227–233.
